# Seasonal variability in the persistence of dissolved environmental DNA (eDNA) in a marine system: The role of microbial nutrient limitation

**DOI:** 10.1371/journal.pone.0192409

**Published:** 2018-02-23

**Authors:** Ian Salter

**Affiliations:** 1 Sorbonne Universités, UPMC Univ Paris 06, CNRS, Laboratoire d’Océanographie Microbienne (LOMIC) Observatoire Océanologique, Banyuls/mer, France; 2 Faroe Marine Research Institute, Torshavn, Faroe Islands; University of Hyogo, JAPAN

## Abstract

Environmental DNA (eDNA) can be defined as the DNA pool recovered from an environmental sample that includes both extracellular and intracellular DNA. There has been a significant increase in the number of recent studies that have demonstrated the possibility to detect macroorganisms using eDNA. Despite the enormous potential of eDNA to serve as a biomonitoring and conservation tool in aquatic systems, there remain some important limitations concerning its application. One significant factor is the variable persistence of eDNA over natural environmental gradients, which imposes a critical constraint on the temporal and spatial scales of species detection. In the present study, a radiotracer bioassay approach was used to quantify the kinetic parameters of dissolved eDNA (*d*-eDNA), a component of extracellular DNA, over an annual cycle in the coastal Northwest Mediterranean. Significant seasonal variability in the biological uptake and turnover of *d*-eDNA was observed, the latter ranging from several hours to over one month. Maximum uptake rates of *d*-eDNA occurred in summer during a period of intense phosphate limitation (turnover <5 hrs). Corresponding increases in bacterial production and uptake of adenosine triphosphate (ATP) demonstrated the microbial utilization of *d*-eDNA as an organic phosphorus substrate. Higher temperatures during summer may amplify this effect through a general enhancement of microbial metabolism. A partial least squares regression (PLSR) model was able to reproduce the seasonal cycle in *d*-eDNA persistence and explained 60% of the variance in the observations. Rapid phosphate turnover and low concentrations of bioavailable phosphate, both indicative of phosphate limitation, were the most important parameters in the model. Abiotic factors such as pH, salinity and oxygen exerted minimal influence. The present study demonstrates significant seasonal variability in the persistence of *d*-eDNA in a natural marine environment that can be linked to the metabolic response of microbial communities to nutrient limitation. Future studies should consider the effect of natural environmental gradients on the seasonal persistence of eDNA, which will be of particular relevance for time-series biomonitoring programs.

## Introduction

The ability to measure species distribution is a fundamental requirement of biodiversity monitoring and conservation biology [1–3]. Environmental DNA (or eDNA) has recently emerged as a powerful tool to study patterns in biodiversity [4] and contribute to marine assessment programs [5,6]. Strictly eDNA can be defined as the total DNA recovered from a specific environment, in which sense it potentially covers intracellular DNA in organisms smaller than the sample, typically microorganisms [7,8], and extracellular or exogenous DNA in organisms larger than the sample [4,9]. Extracellular DNA may include dissolved components, including small mitochondrial organelles, or larger particulate cell fragments. High throughput sequencing of eDNA thus has the potential to describe spatial and temporal patterns in a wide range of organisms [10,11]. Following first successful application in water samples [2], eDNA species detection has been widely applied in aquatic settings: Previous studies have successfully demonstrated the detection of aquatic invertebrates [10,12], reptiles [13], amphibians [10,14], fish [15–20], marine mammals [20,21] and whale sharks [22].

The development of high-throughput sequencing and standardized procedures could facilitate metabarcoding of eDNA to be established in spatially [20] and temporally intensive monitoring of aquatic systems. However, before such approaches can be successfully implemented, an accurate understanding of the factors controlling the release and persistence of eDNA are required [23,24]. Previous studies have addressed eDNA degradation under experimental conditions by examining detection rates following the removal of target organisms. Successful detection of target organisms varies widely, ranging from 0.9–25 days [10,12,15,25,26], depending on the target organism and experimental set-up. Although these studies have highlighted the importance of considering eDNA degradation, it is difficult to extrapolate the findings to natural marine systems. A recent review paper provides a comprehensive discussion of the potential drivers of eDNA in aquatic systems [26]. A significant conclusion from this review is that no study has examined the effect of natural environmental gradients on eDNA degradation. This is particularly relevant considering that in the future eDNA may be applied to time-series biomonitoring of aquatic systems, where environmental gradients likely to influence eDNA degradation are known to vary seasonally.

The fate of DNA in aquatic systems has long been associated with microbial activity [27–35], largely through the action of membrane-bound and extracellular enzymatic activity [36–38]. Abiotic factors such as UV radiation, temperature and pH can directly impact denaturation of DNA [39–43] although together with salinity and dissolved oxygen concentrations [26] may exert an impact indirectly by modulating microbial metabolism and enzyme kinetics [44,45]. Based on the current literature it is reasonable to postulate that in marine systems the relationships between environmental variables and eDNA persistence is predominantly mediated through indirect effects on microbial physiology and enzyme activity. In this context the role of nutrient dynamics is probably of great significance. The microbial breakdown of dissolved organic phosphorous (DOP) pools is known to play a role in phosphorous nutrition under limiting conditions [35,46–48]. Nuclear magnetic resonance studies indicate that 75% of the DOP pool is represented by compounds containing P-ester bonds [49], which includes adenosine triphosphate (ATP) and DNA. To date the concentration of dissolved DNA has been shown to vary seasonally [31], spatially [32] and vertically [34] in the ocean and DNA uptake kinetics have been addressed in nutrient-enriched mesocosm experiments [35,50]. However, the seasonal dynamics of DNA uptake and turnover in response to nutrient limitation, or other environmental factors, remains largely unexplored.

The Mediterranean Sea is considered to be a marine biodiversity hotspot containing approximately 17,000 known species [51]. Shelf waters account for 20% of the sea, compared to 7.6% globally [52]. The coastline is densely populated and expected to reach 175 million by 2025 (UN/MAP/BP/RAC 2005), rendering marine biodiversity particularly sensitive to anthropogenic threats [53] and subject to problems of invasive [54,55] and endangered [56–58] species. However, the absence of robust baselines in the Mediterranean has been identified as a major difficulty in evaluating marine health, thus compromising conservation efforts at the whole ecosystem level [59]. These defining features of the Mediterranean highlight it as a potentially strong beneficiary of recent advances in eDNA surveys. However, the Mediterranean is also a basin characterized by strong environmental gradients [51]. It is a low nutrient basin [60] characterized by west to east gradients in nutrient concentrations, N:P stoichiometry [61,62] and productivity gradients [63,64]. Inorganic phosphate concentrations in the Mediterranean are in the low nanomolar range [61,62] and limit primary productivity [65–67] and bacterioplankton [67–69]. In addition to these spatial gradients, time-series observations in the Northwest Mediterranean also exhibit seasonal gradients in temperature salinity, oxygen, pH, nutrients, phytoplankton biomass and bacterial activity [70–72], all of which are environmental factors thought to impact eDNA persistence [26].

The present work aims to address the paucity of observations in the degradation kinetics of dissolved eDNA (*d*-eDNA), as a component of extracellular DNA, across seasonally occurring environmental gradients in a marine system. Considering the great potential of eDNA to detect a wide range of aquatic organisms, and the possible contribution it can make towards marine policy issues, spatially and temporally intensive eDNA surveys are a likely prospect for the future. However, a better understanding of environmental controls on DNA turnover will be necessary to make the best possible use of future eDNA survey results in marine ecosystems. With these considerations in mind, the main objective of this study was to quantify the seasonal variability in *d*-eDNA turnover in relation to prevailing environmental conditions in the coastal Northwest Mediterranean. Particular emphasis was placed on the importance of nutrient limitation for driving microbial metabolism towards the utilization of *d*-eDNA as a phosphorous substrate.

## Material and methods

### Study site

Samples were collected from a fixed-point time-series site, SOLA, located in the Northwest Mediterranean at 42°31′N, 03°11′E. Station SOLA is a component of the French national coastal observation network SOMLIT (*S**ervice d’**O**bservation en* *M**ilieu* *LIT**toral*). Sample collection and analytical procedures for the analysis of chlorophyll a, dissolved oxygen, pH, dissolved inorganic nutrients, temperature and salinity were carried out according to nationally standardized protocols and data quality procedures of SOMLIT, full details of which are archived at the URL: somlit.epoc.u-bordeaux1.fr. A brief description of these methods is provided below.

### Sample collection

Surface seawater samples were collected bi-weekly from a depth of approximately 3 m. Sample water was collected into acid-washed (10% v/v HCl) polycarbonate carboys from 10 L Niskin bottles attached to a CTD rosette frame. Full depth profiles (25 m) of temperature, salinity and fluorescence were obtained in parallel from a Sea Bird SBE 19 profiler.

#### Chlorophyll

One litre of seawater was collected on a GF/F filter at low pressure (<0.2 bar). Samples were processed immediately after filtration or stored at -20°C for a period < 1 week. Upon processing, samples were soaked in 90% acetone at 4°C for a period of approximately 12–16 h and analyzed within 2 h. Fluorescence was measured before and after acidification to correct for phaeopigments [73,74].

#### Dissolved oxygen

Sampling bottles were washed with 1.2 M HCl, rinsed with deionized water, dried in an oven for 24 h and allowed to equilibrate to room temperature prior to sampling. According to SOMLIT protocols, dissolved oxygen is the first sample to be collected from a Niskin bottle immediately upon recovery. The bottles were rinsed twice with sample water and carefully filled using silicon tubing to avoid turbulence and bubbles. Dissolved oxygen was analyzed by titration following the Winkler method (1988) [74].

#### Dissolved inorganic nutrients

Nitrate, nitrite, phosphate and silicic acid were analyzed by colorimetry [75,76] on a flow-segmented analyzer (SEAL AA3; SEAL analytical). The analysis of unfiltered samples was conducted immediately or on samples stored at -20°C for a maximum period of two months. Calibration was performed using high-purity certified standards (CERTPUR MERCK) of NO_3_ (reference: 1.19811.0500), NO_2_ (1.19899.0500), PO_4_ (1.19898.0500) and Si(OH)_4_ (1.12310.0500) prepared in a medium of ultra-pure artificial seawater. Detection limits were 0.05 μmol L^-1^ for NO_3_+NO_2_, 0.003 μmol L^-1^ for Si(OH)_4_ and 0.006 μmol L^-1^ for PO_4_.

#### Temperature and salinity

A Seabird SBE 19 sensor was used to acquire continuous measurements of conductivity, temperature and depth during CTD casts. The SBE 19 device is returned to Seabird annually for calibration. Minimum precision for the measurements was 0.01°C for temperature and 0.01 units of salinity (or 0.05% of the measurement value). Raw CTD data were typically binned at 0.25 m depth intervals.

#### Bacterial production

The incorporation of ^3^H-leucince was measured using the centrifugation method [77]. Subsamples (1.5 mL; three replicates and two blanks killed with 50% of trichloracetic acid (TCA)) were incubated for 2 h in the dark at *in situ* temperature with a mixture of ^3^H-leucine (Perkin Elmer, (SA) 115.4 Ci mmol^−1^) and non-radioactive leucine at final concentrations of 7 and 13 nM, respectively. Incubations were stopped by the addition of TCA to a final concentration of 5%. After a centrifugation step at 13,300 x g for 15 minutes the supernatant was discarded and 0.5 mL of 5% TCA were added. This step was performed twice with a second centrifugation for 5 minutes. Ethanol (0.5 mL of 70%) was added prior to the last centrifugation step of 5 minutes. The supernatant was discarded and 1 mL of PCS liquid scintillation cocktail was added. The radioactivity incorporated into bacterial cells was measured with a LS 6500 Beckman liquid scintillation counter.

### Phosphate, ATP and DNA kinetics

The kinetics of orthophosphate, adenosine triphosphate (ATP), and deoxyribonucleic acid (DNA) uptake were measured by the Rigler bioassay approach [78] using a concentration series bioassay [35,79]. Phosphate and ATP bioassays were carried out using commercially available isotopes (H_3_^33^PO_4_, AT-γ^33^P) and radiolabeled DNA was synthesized as described below.

#### Radiolabelling of DNA

Radiolabelling of DNA follows the protocol of Løvdal et al. 2007. Random oligonucleotide primed synthesis was carried out using the Decalabel DNA labeling kit (Fermentas; K0621). This procedure results in products of variable lengths, here the average length was estimated to be 0.48 kbp according to an experimentally derived equation [80]. Lambda DNA HindIII digests (Fermentas; SM0101) were used as DNA templates and deoxyadenosine-[α-^33^P]-triphosphate (3000 Ci mmol^-1^ / 111 TBq mmol^-1^; Perkin Elmer—NEG312H250UC) in 10 mL of tricine as the radioactive precursor. The half-life of phosphorous-33 is 25.4 days. A freshly-labeled DNA product was prepared at the beginning of each month throughout 2012 to ensure it was used within one half-life of ^33^P and to avoid significant shortening of the DNA chain length by radiochemical decay. Prior to use, primers and unincorporated deoxynuceotide triphosphates were removed through purification with a Genejet centrifugal filter device (Fermentas). The DE-81 filter-binding assay was used to determine typical label incorporation, DNA yield and specific activity of the purified product [81]. The radiolabelled DNA had label incorporation >98% with a specific activity ranging from 3.5 x 10^7^–6.7 x 10^8^ cpm μg^-1^.

#### Bioassay incubations

Samples were collected in 1 L acid-washed thermos flasks [79] to maintain *in situ* temperatures until rate measurements could begin, which occurred within 30 minutes of sample collection. Carrier-free ^33^PO_4_^-3^ (as H_3_^33^PO_4_; 155.8 Ci mg^-1^, 5.76 TBq mg^-1^, Perkin elmer—NEZ080) and gamma labeled ATP (Adenosine 5’-Triphosphate, [γ -33P]: 3000 Ci mmol^-1^, 111 Tbq mmol^-1^, Perkin Elmer—NEG302H) were diluted in ultra-pure water (0.2 μm sterile-filtered and autoclaved milli-Q) to create working solutions of 10.1 nM. The radiolabelled DNA product was diluted in ultra pure water to create a working solution of 10.1 ng mL^-1^. For the standard addition bioassay experiments, 40 μL of each working solution was added to 4 mL of sample seawater in 5 mL sterile cryotubes to produce final tracer concentrations of 0.1 nM for ^33^PO_4_^-3^ and AT-γ^33^P and 0.1 ng mL^-1^ for DNA-^33^P, corresponding to <10% of anticipated *in situ* concentrations [35]. For the phosphate and ATP incubations, samples were diluted with non-labeled standards to create a dilution series of 0, 4, 8, 12, 24, 48, 96, and 192 nM. A non-labeled DNA standard was created by diluting DNA III Hind stock in ultrapure water and was added to samples to create a dilution series of 0, 2, 4, 8, 12, 25, 50, 100 ng mL^-1^. Samples were incubated at *in situ* temperatures in a temperature controlled incubation chamber.

All incubations were terminated after 15–60 minutes through the addition of 40 μL of a cold chase cocktail containing 100 mM of PO_4_^-3^, 100 mM of ATP and 50,000 μg L^-1^ of DNA resulting in final incubation concentrations of 1 mM PO_4_^-3^ and ATP and 500 μg L^-1^ of DNA. Cold chase saturation [82] was favored over PFA fixation [79] to reduce the loss of incorporated tracer upon fixation. Blank incubations were performed identical to samples with the exception that the cold chase cocktail was added 15 minutes prior to the radioactive tracers. Upon completion of the incubation, blanks and samples were gently filtered (< 100 mbar vacuum) onto 25 mm 0.2 μm polycarbonate membranes (Millipore) backed with GF/C filters (Whatman). Prior to use, all membrane and glass fibre filters were soaked overnight in a 10 mM phosphate solution to minimize binding of unincorporated radioactive tracers during filtration. Membrane filters from ^33^PO_4_^-3^ and ATP incubations were rinsed with 3 mL of <0.2 μm-filtered sample water. DNA-^33^P filters were rinsed with 3 mL of <0.2 μm-filtered sample water amended with low molecular weight salmon sperm (Sigma Aldrich) at a concentration of 10 μg mL^-1^. After filtration polycarbonate membrane filters were transferred to 6 mL pony vials and 4 mL of Ultima Gold MV scintillation cocktail was added. Radioactivity on the filters was measured as counts per minute (cpm) with a LS 6500 Beckman liquid scintillation counter. Turnover was calculated according to Eqs 1 and 2

$$T_{0}=t/(-\text{ln}\left(1-R\right)$$ (1)

$$R=\left({\text{S}}_{\text{cpm}}-{\text{B}}_{\text{cpm}}\right)-{\text{T}}_{\text{cpm}}$$ (2)

**Fig 1:**
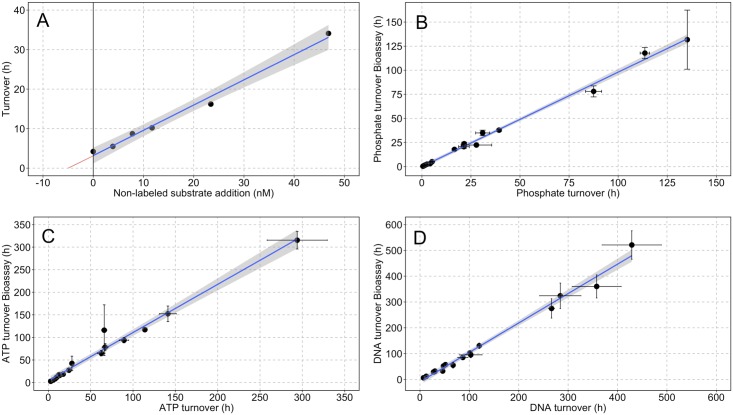
Radioactive bioassay concentration series. Panel (A) provides an example of the Rigler bioassay approach [78] using ^33^PO_4_^3-^ uptake data from 04.04.2012. The turnover of ^33^PO_4_^3-^ (*T*_*0*_) was measured over a concentration series of non-labeled substrate additions (S*a*). The Lineweaver-Burk plot showed here is a linear representation of Michaelis-Menten uptake kinetics, whereby the slope of the regression (1/V_max_) provides an estimate of substrate uptake rate, the negative intercept on the x-axis (*k* + S*n*: red line) is an estimate of the natural substrate concentration (S*n*) at ambient conditions (S*a* = 0), where *k* is the transport constant and assumed to be negligibly small [79], and the y-intercept is substrate turnover (*k* + Sn / V_max_) at zero added substrate (S*a* = 0). In the case of phosphate, *k* + S*n* is taken as an estimate of biologically available phosphate and distinct from soluble reactive phosphorous (SRP) determined analytically. Panels (B-D) provide verification of the substrate uptake models by comparing (*k* + Sn / V_max_) with turnover rates (*T*_*0*_) measured from independent experiments. Error bars on measured turnover rates (x-axis) represent ± 1 σ from n = 3 replicates and error bars on bioassay estimates (y-axis) are estimated from the LINEST function of the concentration series (n>4). Regression statistics (slope ± standard error; p-value; r^2^; F-statistic; degrees of freedom, p-value) for ^33^PO_4_^3-^ (1.01 ± 0.02; <0.001; 1.00; 2561; <0.001), AT^33^P (0.96 ± 0.01; <0.001; 1.00, 1.08 x 10^4^; 16, <0.001) DNA-^33^P (1.02 ± 0.04; <0.001; 0.98. 671.3; 16; 0.001).

Where *T*_*0*_ is the substrate turnover (h), *t* is the incubation time (h), ln is the natural logarithm and S_cpm_, B_cpm_ and T_cpm_ are the radioactive counts of the samples, experimental blanks and tracer additions, respectively. Turnover was plotted against the concentration of non-labeled substrate additions (S*a*) to produce a linear representation (Lineweaver-Burk) of Michaelis-Menten substrate kinetics (Fig 1B and 1C). The y-intercept on the Lineweaver-Burk plot was used to calculate substrate turnover at ambient concentration (S*n*) (Fig 1A). Triplicate turnover measurements with no added substrate (S*a* = 0) were performed as an independent verification of the kinetic model and to validate the assumption that the transport constant (*k*) is negligibly small compared to ambient substrate concentrations [79]. The LINEST statistical function was used to determine standard errors on substrate uptake rates (V_max_), ambient substrate concentrations (*k* + S*n*) and turnover at ambient concentrations ((*k* + S*n*) / V_max_).

### Partial least squares regression (PLSR) model

A statistical model to predict turnover of *d*-eDNA was developed based on projection to latent structures using PLSR. PLSR was selected as a predictive tool as it negates the assumptions of zero co-linearity in explanatory variables that characterize linear regression models, which is typically significant in environmental datasets [72]. PLSR linearly extracts a few latent variables that are most useful in modeling the response. The model was used to predict a single y–value (measured turnover of *d*-eDNA) from a set of environmental parameters (temperature, salinity, day length, pH, dissolved oxygen, dissolved inorganic nitrate + nitrite, biologically available phosphorous, and phosphate turnover). The model was constructed using the seasonal dataset of approximately bi-weekly resolution. Input data were scaled to standardized values (mean = 0, variance = 1). The number of PLSR components was chosen by examining minima in root mean squared error of prediction (RMSEP) values following k-fold cross validation (k = 10) and cross-validated r^2^ values, the latter taken as a measure of the PLSR model’s goodness of fit [83]. The PLSR model was optimized with an r^2^ value of 0.60 using two components (S1 File). Standardized regression coefficients were calculated for each predictor variable to ascertain the most important measured environmental parameters explaining the variance in microbially-mediated *d*-eDNA turnover. All analyses were performed in the R packages PLS and plsdepot [84] and the code is archived in the supporting information (S2 File).

## Results

### Environmental conditions

**Fig 2:**
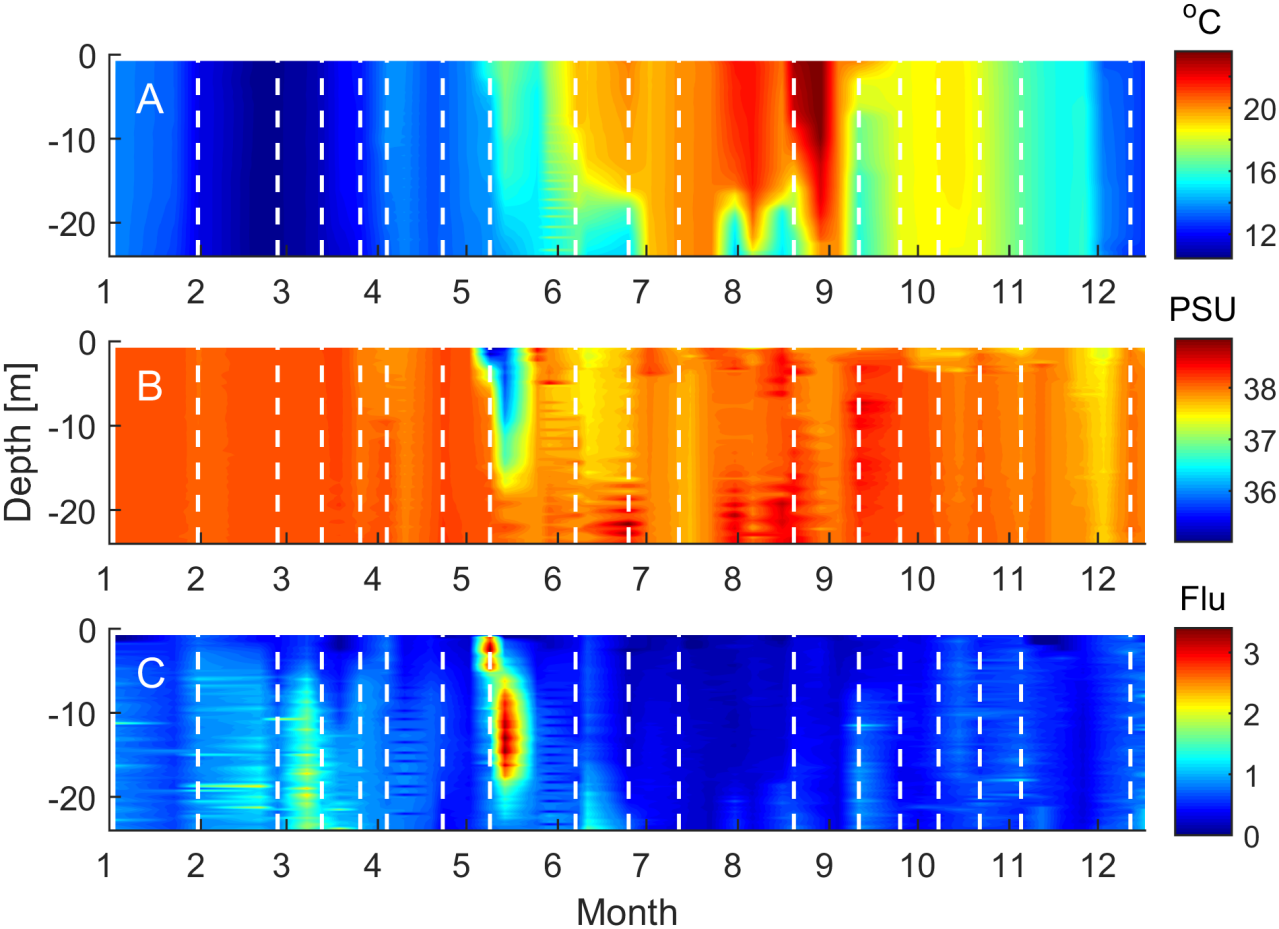
Depth profiles of environmental conditions. Full depth profiles of (A) temperature, (B) salinity and (C) fluorescence were measured at station SOLA in 2012. White dashed lines mark the dates when discrete samples were taken from the surface (3 m) to carry out phosphorous bioassay experiments (Solid black squares in Figs 3 and 4).

**Fig 3:**
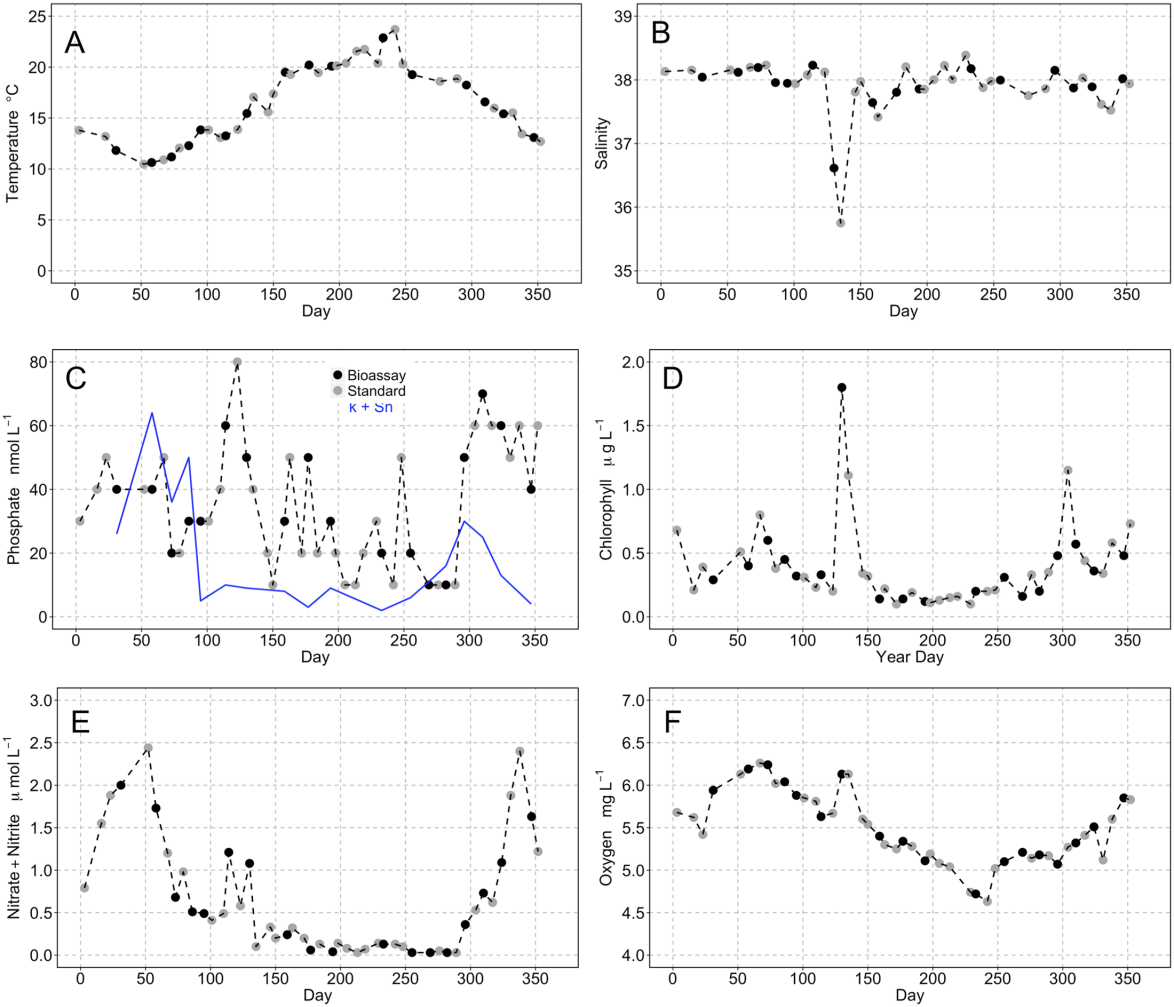
Time series observations of environmental parameters. Time series data of environmental parameters monitored in parallel to bioassay experiments during 2012 for (A) temperature, (B) salinity, (C) inorganic phosphate (PO_4_^3-^), (D) chlorophyll, (E) dissolved inorganic nitrogen (DIN = NO_3_^2-^ + NO_2_^-^) and (F) dissolved oxygen. In panel (C) the dashed line is soluble reactive phosphorous (SRP) determined analytically and the solid blue line is an estimate of biologically available phosphate (*k* + S*n*). Grey symbols mark the sampling dates when standard environmental parameters were measured and black symbols mark those where the kinetic bioassay experiments were conducted.

Full depth profiles of temperature, salinity and fluorescence were obtained with weekly resolution from CTD casts (Fig 2). The minimum temperature observed at 3 m from the CTD data was 10.6°C on 27.02.2012 rising progressively to a maximum of 23.7°C on 29.08.2012 (Fig 2A). Discrete samples for incubation work were collected on the 27.02.2012 and 20.08.2012, corresponding to the maximum seasonal temperature range of 12.2°C (Fig 3A). Surface salinity values were relatively constant throughout the year, fluctuating from 37.4 to 38.4 PSU with no clear seasonality. An episodic reduction in salinity occurred between the 9^th^ and 14^th^ May with surface values dropping to 35.7 PSU (Fig 3B). This low salinity feature penetrated to a depth of approximately 15 m in the water column (Fig 2B) and corresponded to significantly enhanced water column fluorescence values (Fig 2C). Surface chlorophyll values peaked at 1.8 μg L^-1^ on the 9^th^ May in association with this low salinity feature (Fig 3D). Aside from these exceptionally high values, surface chlorophyll peaked at 0.8 μg L^-1^ on the 7^th^ March and 1.15 μg L^-1^ on 30^th^ October and were <0.2 μg L^-1^ for a 90 day period from 07.06—04.09.2012.

Values of soluble reactive phosphorous (SRP) fluctuated between 0.03 and 0.06 μM for most of the year with summer values frequently at, or close to the detection limit of 0.01 μM (Fig 3C). Bioavailable phosphorous concentrations (*k* + S*n*), calculated from bioassay incubations, exhibited a more pronounced seasonality. Concentrations were between 0.03 and 0.06 μM from January through to March and then fell sharply and remained <0.01 μM until the end of September. Bioavailable phosphorous concentrations exhibited a secondary peak of 0.03 μM on 26.10.2012. Dissolved inorganic nitrogen concentrations (DIN) showed similar seasonality to bioavailable phosphorous (Fig 3E). Peak values of 2.5 μM were measured in early spring (21.02.2012) and early winter (03.12.2012), with significant depletion (<0.3 μM) observed between the 25^th^ May and 15^th^ October. Dissolved oxygen concentrations displayed a clear seasonal pattern with highest values >5.5 mg L^-1^ recorded in spring and lowest values (<5 mg L^-1^) during summer (Fig 3F). Peak values of 6.13 mg L^-1^ were observed on the 9^th^ and 14^th^ May and associated to the episodic reduction in salinity (Figs 2B and 3B).

### Isotope dilution experiments

The kinetic parameters and associated statistics calculated from the radioactive substrate bioassays are summarized in Tables 1–3. The substrate uptake data was well described by a linear representation of Michealis-Menten kinetics with r^2^ values of 0.93–1.00, 0.85–1.00, and 0.77–0.99 for PO_4_^-3^, ATP and DNA, respectively (Tables 1–3). An independent assessment of turnover time (*T*_0_) with no added substrate (S*a* = 0) was positively correlated to turnover at natural substrate concentrations (S*n*) estimated from the linear model (*k +* S*n* / V_max_) for PO_4_^-3^ (r^2^ = 0.98; Fig 1B), ATP (r^2^ = 0.99; Fig 1C) and DNA (r^2^ = 0.98; Fig 1D) and approximated a 1:1 relationship; slopes = 1.01 ± 0.02 (PO_4_^-3^), 0.96 ± 0.01 (ATP) and 1.02 ± 0.04 (DNA) (Fig 1).

**Table 1:** Summary of sampling dates and inorganic phosphate (PO_4_^3-^) kinetic parameters estimated from the Rigler bioassay approach. The r^2^ values are based on the regression statistics of substrate turnover against the concentration series of non-labeled substrate additions.

Sampling Date	V_max_ (nmol L^-1^ h^-1^)	*k +* S*n* (nmol L ^-1^)	(*k +* S*n*) / V_max_ (h)	*T*_*0*_ (h)	r^2^
31/01/12	0.22 ± 0.02	26.1 ± 2.81	117.8 ± 5.82	113.6 ± 2.41	0.98
27/02/12	0.82 ± 0.09	63.6 ± 8.38	78.0 ± 5.76	87.5 ± 4.11	0.93
13/03/12	1.52 ± 0.17	35.9 ± 4.66	23.7 ± 1.64	21.6 ± 1.16	0.96
26/03/12	2.22 ± 0.24	49.7 ± 5.49	22.4 ± 0.42	22.8 ± 7.79	0.98
04/04/12	1.56 ± 0.08	4.96 ± 1.17	3.17 ± 0.73	4.21 ± 0.12	0.99
23/04/12	0.50 ± 0.08	10.4 ± 1.86	20.7 ± 1.63	21.4 ± 2.73	0.97
09/05/12	n.d.	n.d.	n.d.	1.25 ± 0.04	n.d.
07/06/12	8.49 ± 0.28	8.40 ± 0.51	0.99 ± 0.05	0.97 ± 0.05	0.99
25/06/12	6.50 ± 0.20	2.77 ± 0.41	0.43 ± 0.06	0.53 ± 0.03	0.99
12/07/12	3.60 ± 0.05	9.19 ± 0.28	2.55 ± 0.07	2.64 ± 0.09	0.99
20/08/12	3.57 ± 0.12	2.22 ± 0.24	0.62 ± 0.06	0.74 ± 0.01	0.99
11/09/12	3.46 ± 0.07	5.68 ± 0.18	1.64 ± 0.04	1.66 ± 0.03	1.00
25/09/12	2.07 ± 0.05	10.7 ± 0.63	5.14 ± 0.28	5.21 ± 0.09	1.00
08/10/12	0.90 ± 0.03	16.1 ± 0.89	17.8 ± 0.83	16.6 ± 0.44	0.99
22/10/12	0.79 ± 0.05	30.1 ± 2.19	37.8 ± 1.09	39.3 ± 0.63	0.97
05/11/12	0.72 ± 0.09	25.3 ± 3.73	35.0 ± 2.83	31.0 ± 3.58	0.96
19/11/12	0.14 ± 0.03	13.3 ± 4.39	92.0 ± 24.6	n.d.	0.96
12/12/12	0.03 ± 0.002	4.32 ± 1.05	131.7 ± 30.7	131.7 ± n.d.	0.99

**Table 2:** Summary of sampling dates and ATP kinetic parameters estimated from the Rigler bioassay approach. The r^2^ values are based on the regression statistics of substrate turnover against the concentration series of non-labeled substrate additions.

Sampling Date	V_max_ (nmol L^-1^ h^-1^)	*k +* S*n* (nmol L^-1^)	(*k +* S*n*) / V_max_ (h)	*T*_*0*_ (h)	r^2^
31/01/12	0.02 ± 0.003	2.24 ± 1.13	116.0 ± 56.1	66.0 ± 3.15	0.95
27/02/12	0.10 ± 0.01	4.21 ± 1.67	42.4 ± 16.1	27.9 ± 2.07	0.97
13/03/12	0.22 ± 0.03	17.5 ± 3.08	78.4 ± 8.04	67.1 ± 3.57	0.91
26/03/12	0.25 ± 0.05	16.3 ± 3.66	64.9 ± 6.07	62.5 ± 4.46	0.92
04/04/12	1.70 ± 0.03	19.9 ± 2.29	11.7 ± 1.02	9.90 ± 0.46	0.98
23/04/12	0.08 ± 0.03	12.3 ± 2.76	152.3 ± 17.2	141.2 ± 9.89	0.93
09/05/12	n.d.	n.d.	n.d.	2.80 ± 0.26	n.d.
07/06/12	0.95 ± 0.16	11.2 ± 2.04	11.8 ± 0.68	9.98 ± 1.14	0.89
25/06/12	1.01 ± 0.17	8.32 ± 1.43	8.25 ± 0.34	8.08 ± 0.07	0.92
12/07/12	0.32 ± 0.08	5.10 ± 1.28	15.83 ± 1.49	13.2 ± 0.16	0.85
20/08/12	0.94 ± 0.07	5.23 ± 0.46	5.58 ± 0.29	5.67 ± 0.06	0.97
11/09/12	0.48 ± 0.04	8.33 ± 1.23	17.5 ± 2.20	12.7 ± 0.30	0.97
25/09/12	0.04 ± 0.004	1.10 ± 0.15	27.3 ± 2.71	24.3 ± 0.31	0.98
08/10/12	0.03 ± 0.002	2.39 ± 0.23	93.9 ± 5.92	89.4 ± 5.60	0.98
22/10/12	0.01 ± 0.003	1.83 ± 0.14	18.8 ± 1.27	17.5 ± 0.40	1.00
05/11/12	0.04 ± 0.002	4.30 ± 0.29	117.1 ± 3.40	114.0 ± 1.44	0.99
19/11/12	0.01 ± 0.002	3.07 ± 0.54	315.4 ± 19.6	293.9 ± 35.6	0.94
12/12/12	0.03 ± 0.003	42.5 ± 3.78	1264.3 ± 34.3	1218.2 ± 339.3	0.97

**Table 3:** Summary of sampling dates and DNA kinetic parameters estimated from the Rigler bioassay approach. The r^2^ values are based on the regression statistics of substrate turnover against the concentration series of non-labeled substrate additions.

Sampling Date	V_max_ (nmol L^-1^ h^-1^)	*K + Sn* (nmol L^-1^)	(*k +* S*n*) / V_max_ (h)	*T*_*0*_ (h)	r^2^
31/01/12	0.028 ± 0.007	14.6 ± 3.89	521.0 ± 55.2	428.9 ± 60.2	0.85
27/02/12	0.090 ± 0.01	4.95 ± 1.10	55.4 ± 11.3	67.0 ± 2.62	0.97
13/03/12	0.060 ± 0.009	5.37 ± 1.10	95.3 ± 11.1	102.8 ± 23.3	0.95
26/03/12	0.18 ± 0.008	9.12 ± 0.66	57.3 ± 3.1	51.6 ± 1.47	0.99
04/04/12	0.09 ± 0.007	8.87 ± 0.82	103.0 ± 5.46	101.2 ± 3.07	0.98
23/04/12	0.03 ± 0.008	9.28 ± 3.14	360.3 ± 44.8	357.8 ± 49.8	0.83
09/05/12	4.47 ± 0.46	28.8 ± 3.15	6.43 ± 0.25	7.03 ± 0.12	0.96
07/06/12	0.25 ± 0.01	7.19 ± 0.48	28.9 ± 1.39	27.5 ± 1.75	0.99
25/06/12	0.22 ± 0.03	7.28 ± 1.30	32.6 ± 4.37	45.9 ± 0.73	0.94
12/07/12	0.53 ± 0.09	16.3 ± 3.21	30.9 ± 2.78	30.1 ± 1.99	0.86
20/08/12	0.81 ± 0.12	9.93 ± 1.62	12.2 ± 0.75	12.7 ± 0.45	0.77
11/09/12	0.08 ± 0.002	6.60 ± 0.21	85.2 ± 2.23	86.8 ± 10.4	0.99
25/09/12	0.01 ± 0.001	3.02 ± 0.51	275.1 ± 37.3	266.4 ± 6.14	0.96
08/10/12	0.01 ± 0.001	3.68 ± 0.64	324.1 ± 49.3	284.1 ± 42.1	0.98
22/10/12	0.31 ± 0.02	16.3 ± 1.18	51.9 ± 2.67	47.5 ± 2.37	0.98
05/11/12	0.09 ± 0.01	12.1 ± 1.63	130.5 ± 10.7	120.0 ± 3.79	0.95
19/11/12	0.46 ± 0.07	14.8 ± 2.51	32.3 ± 2.09	30.3 ± 2.64	0.91
12/12/12	0.001 ± 0.0001	0.90 ± 0.27	794.4 ± 224.2	889.4 ± 20.4	0.99

**Fig 4:**
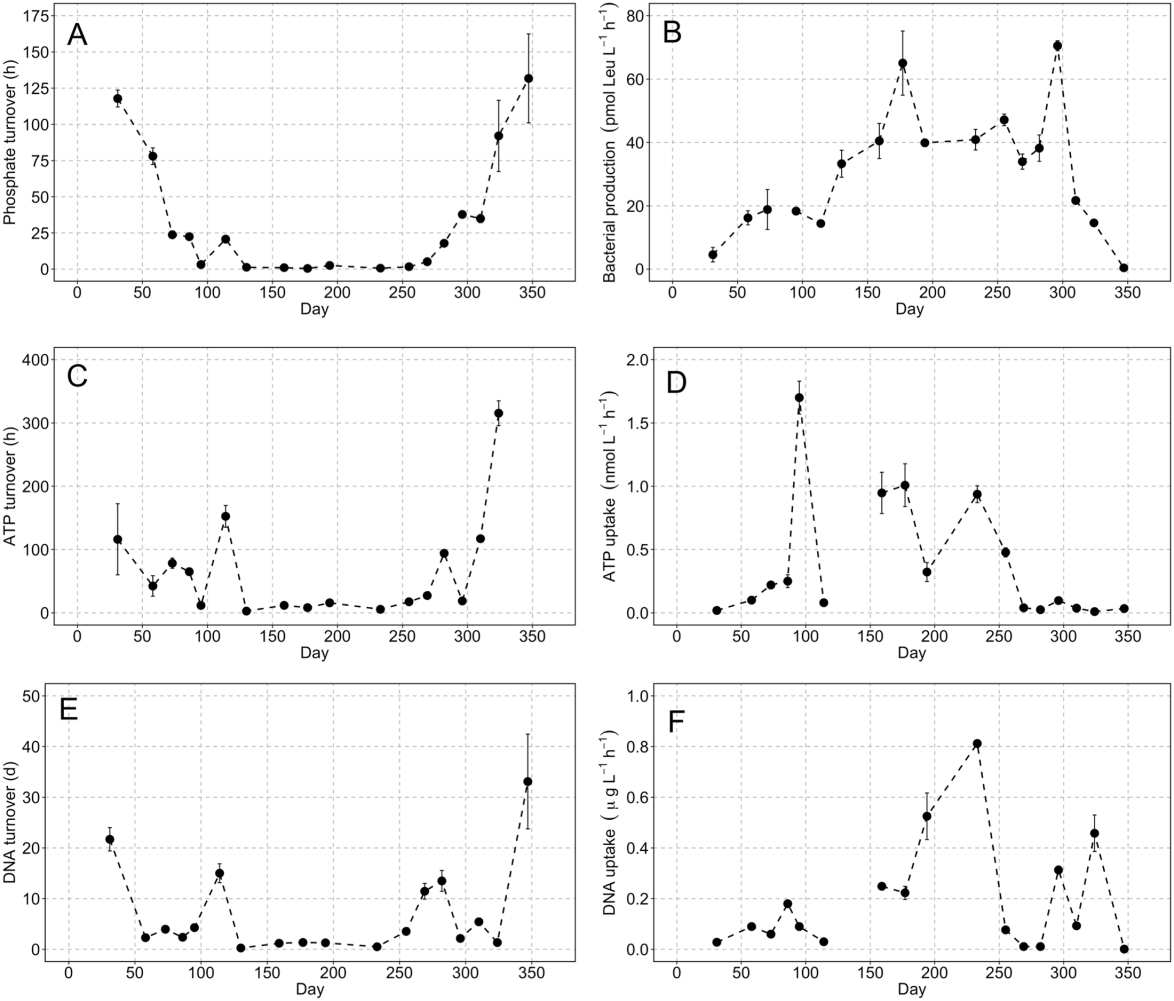
Time-series data of substrate uptake and turnover kinetics. (A) Inorganic phosphate turnover, (B) ATP turnover, (C) ATP uptake, (e) DNA turnover and (F) DNA uptake. Error bars are computed from the LINEST function of the concentration series bioassay data (Tables 1–3). Panel (B) shows community-level leucine incorporation and is taken as an estimate of bacterial production [77]; error bars are from replicate incubations (n = 3). Grey symbols mark the sampling dates when standard environmental parameters were measured and black symbols mark those where the kinetic bioassay experiments were conducted.

The turnover of PO_4_^-3^ showed a strong seasonal pattern with values steadily decreasing from 118 h in January to 1.2 h in May (Fig 4A). The turnover of phosphate remained < 6 h for a five month period during summer, starting to rise again on the 8^th^ October and reaching a maximum winter value of 131 h on 12^th^ December. The turnover of DNA (Fig 4E) exhibited similar seasonality with an extended period of high turnover, ranging from 6–85 h, occurring between 9^th^ May and 7^th^ September. The pattern was comparable for ATP with turnover times of 3–27 h over the same period (Fig 4C). The high turnover times of ATP and DNA found during summer months correspond to periods of enhanced biological uptake; ATP V_max_ values of 0.32 ± 0.08 to 1.01 ± 0.17 nmol L^-1^ h^-1^ (Fig 4D) and DNA V_max_ values of 0.22 ± 0.03 to 4.47 ± 0.46 ng L^-1^ h^-1^ (Fig 4F) were measured between May and September (Tables 1–3). The highest DNA uptake rate of 4.47 ± 0.46 ng L^-1^ h^-1^ occurred on 9^th^ May corresponding to a salinity minimum of 36.6 PSU (Fig 3B) and the lowest observed DNA turnover of 7.03 ± 0.12 h (Table 3). Leucine incorporation rates were highest during summer months peaking at 70.5 pmol Leu. L^-1^ h^-1^ on the 22^nd^ October.

### Phosphate limitation categories

**Fig 5:**
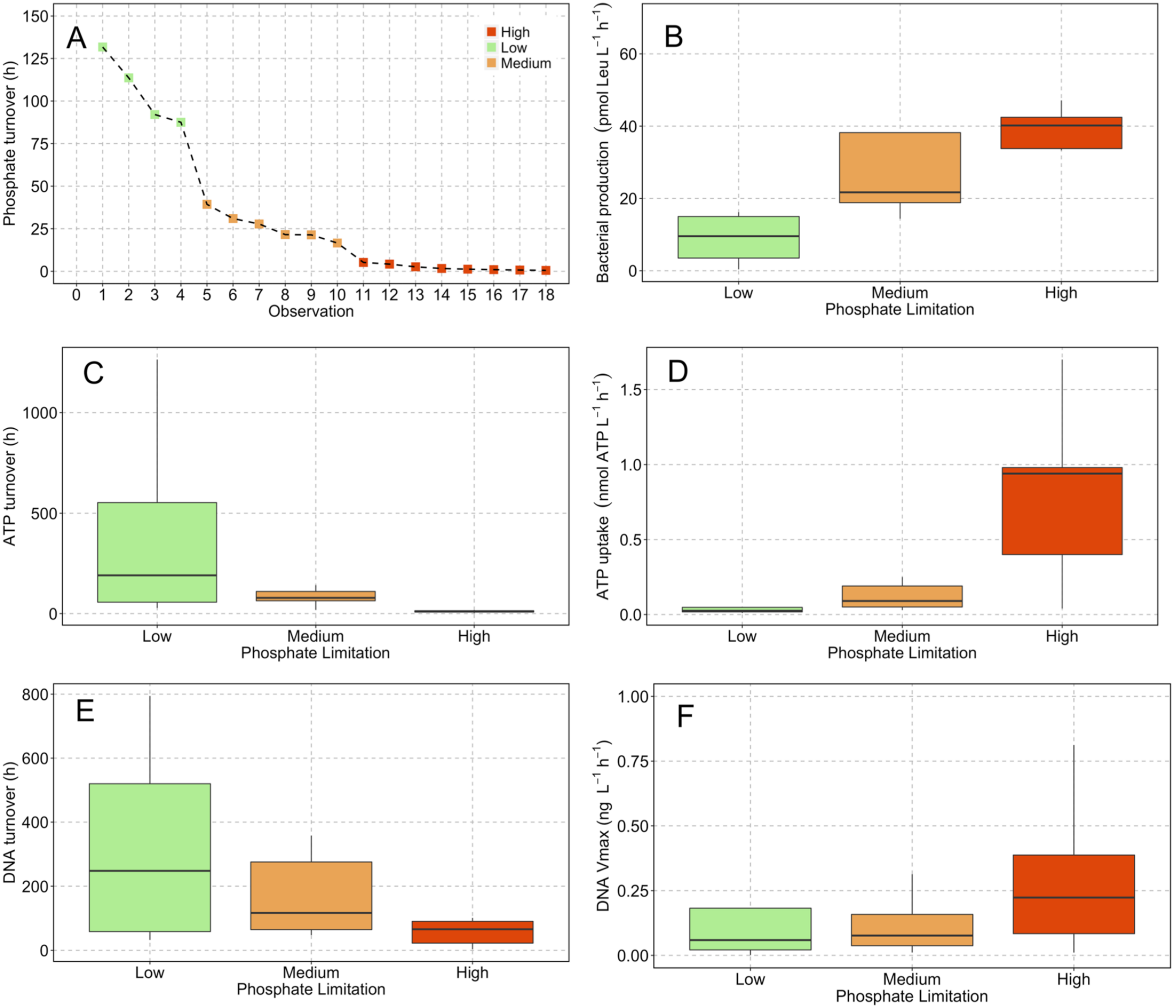
Phosphate limitation categories. Panel (A) shows the empirically derived phosphate limitation categories. Category limits were defined on the basis of successive values increasing by a factor ≥2 and constrained by ensuring that differences between the mean of each category were statistically significant resulting in three categories; high, medium and low phosphate limitation. Panels (B-F) are box and whisker plots of kinetic uptake within these empirically defined categories. Statistically significant differences between the categories are summarized in Table 4.

Phosphate limitation categories were determined by ranking all measures of PO_4_^-3^ turnover in descending order (Fig 5A). Categories were subsequently defined on the basis of successive values increasing by a factor ≥2 and constrained by ensuring that differences between the mean of each category were statistically significant. This approach resulted in three categories; high, medium and low phosphate limitation with turnover times of 0.5–5 h, 16–40 h and 90–131 h, respectively (Fig 5A). All permutations of category subset comparisons had means that were statistically different to each other (Welch two sample t-test) at significance levels ≥ P < 0.005 (Table 4).

**Table 4:** Statistical significance of differences in kinetic parameters between phosphate limitation categories. A summary of the results from Welch two sample t-tests comparing the arithmetic means of kinetic parameters between phosphate limitation categories (Fig 5).

Parameter	Phosphate limitation categories		
	High vs. Low	High vs. Medium	Medium vs Low
^33^PO_4_^3-^ (*T*_*0*_)	<0.001	<0.005	<0.001
AT^33^P (*T*_*0*_)	<0.05	n.s.	<0.005
^33^P-DNA (*T*_*0*_)	<0.05	n.s.	<0.10
Leucine (V_max_)	<0.025	<0.10	n.s.
AT^33^P (V_max_)	<0.05	<0.10	<0.025
^33^P-DNA (V_max_)	n.s. (<0.10)*	n.s.	n.s.

*Results of t-test with DNA outlier removed. See text for further explanation.

The turnover time of ATP and DNA decreased progressively from low to high phosphate limitation (Fig 5C and 5E). The median decreased from 190>78>9.9 h for ATP turnover and from 248>117>66 h for DNA. Comparison of the arithmetic means (Welch two sample t-test) showed that turnover times between high-low and medium-low phosphate limitation categories are statistically significant (Table 4). Bacterial production rates increased from low to high phosphate limitation (Fig 5B) with median values of 9.6>21.7>40.2 pmol Leu. L^-1^ h^-1^. The arithmetic means were significantly different in the high-low and high-medium comparison (Table 4). The median values of V_max_ uptake were comparable between low and medium categories: 0.03 cf. 0.09 nmol L^-1^ h^-1^ for ATP and 0.06 cf. 0.08 μg L^-1^ h^-1^ for DNA (Fig 5D and 5E). However, the median V_max_ uptake during high phosphate limitation was notably higher, 0.94 nmol L^-1^ h^-1^ and 0.23 μg L^-1^ h^-1^ for ATP and DNA, respectively. The difference in means for ATP V_max_ was statistically different for each category. The DNA V_max_ dataset was characterized by two extreme values in both the low and high phosphate limitation categories that generate standard errors of 148 and 186%, in part related to the extreme salinity anomaly on 9^th^ May (Fig 3B), for which ATP V_max_ uptake data is not available. These values were identified as outliers by comparing them to the difference between the interquartile range (IQR) and 3^rd^ quartile. Typically if this value is greater than 1.5 a data point can be identified as an outlier in a statistically robust framework. In the case of the DNA V_max_ outliers, they had values of 2.9 and 8.8 for low and high phosphate limitation categories, respectively, and were excluded on this statistical basis resulting in a difference between the arithmetic mean of the high and low phosphate limitation (Table 4).

### Partial least squares regression model

**Fig 6:**
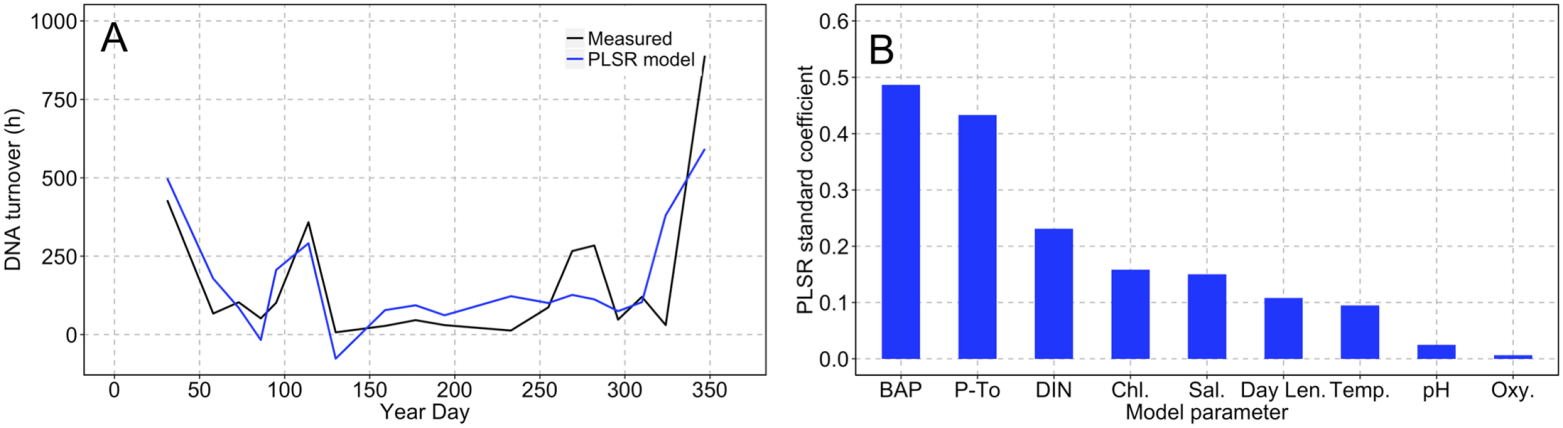
Prediction of dissolved eDNA turnover from environmental parameters. Results from partial latent squares regression (PLSR) model. Panel (A) shows the annual time series of measured *d*-eDNA turnover (black line) and PLSR model output (blue line). Linear regression statistics are slope ± standard error (1.00 ± 0.20), p-value (<0.0005), r^2^ (0.60), F-statistic (24.79), degrees of freedom (16), p-value (<0.0005). Panel (B) shows un-signed standardized model coefficients of the environmental parameters in the PLSR model: BAP (biologically available phosphate (*k* + S*n*); Figs 1A and 3C), P-*T*_*0*_ (phosphate turnover), DIN (dissolved inorganic nitrogen; nitrate + nitrite), Chl. (chlorophyll), Sal. (salinity), Day Len. (day length), Temp. (temperature), pH and Oxy (dissolved oxygen).

The turnover of *d*-eDNA determined from the kinetic bioassay experiments was combined with measured environmental parameters to develop a model that described seasonal *d*-eDNA persistence based on PLS regression analysis (Fig 6). The number of components was selected by examining minimum RMSEP values from 10-fold cross validation and cross-validated r^2^ values as measure of goodness of fit to the observed data (S1 File). The model was optimized with two components that described 60% of the observed variance in *d*-eDNA turnover (Fig 6A). Biologically available phosphorous and phosphate turnover, both taken as measures of the degree of phosphate limitation, were the most important variables in the model (Fig 6B).

## Discussion

The present study demonstrates a strong seasonal component in the turnover of dissolved environmental DNA (*d*-eDNA), ranging from 3 h to > 1 month, in relation to variable environmental conditions. An important consideration of implementing eDNA as a biomonitoring tool concerns the temporal and spatial scales over which the observations can be considered as representative [24,43]. In a dynamic fluid environment, e.g. the coastal ocean, temporal and spatial boundaries are ill defined in comparison to terrestrial ecosystems Consequently the positive detection of a species with the eDNA approach in aquatic settings may represent one of the following: (i) presence of the species at a given site at the time of sampling, (ii) presence of the species at the sampling site from some time previous, or (iii) presence of the species at another location and time; whereby the eDNA signature has been introduced to the sampling site through physical processes such as advection and vertical mixing. The degradation, or turnover time, of eDNA from its point of production is critical in this regard. Although the degradation of eDNA has been recognized as an important constraint on its application [26], and has been addressed in several experimental proof of concept studies [24,42,43], it has not been explicitly considered in natural settings or in the context of seasonal variability. The present findings therefore have important implications when considering the use of eDNA and next generation sequencing approaches to sustained monitoring of biodiversity in marine waters [85], especially when applied to large spatial gradients [20] or time-series observations.

The coastal study site in the Northwest Mediterranean that was the focus of this present study is a phosphorous limited system. Mineral phosphate concentrations, analytically determined as soluble reactive phosphorous (SRP), were < 70 nM and dissolved inorganic nitrogen to phosphate ratios were in excess of Redfield values (median: 28.8); comparable to the Northwest Mediterranean basin as a whole [61,62]. Intuitively phosphate limitation should increase following the spring bloom, however little seasonal variation in SRP concentrations was observed. It has been shown previously that in P-limited environments SRP concentrations over-estimate mineral phosphate concentrations [79], due in part to the acidic conditions of the molybdate reaction that may hydrolyze labile organic phosphorous compounds. Bioavailable phosphate concentrations were estimated from the Rigler bioassay approach and yielded values that were typically lower than the SRP measurements. Concentration data may obscure variability in the rate a particular nutrient is cycled, especially at low concentrations characterizing nutrient limited systems. In this regard, phosphate turnover is generally considered a more dynamic metric of P-limitation [86]. In the present study both phosphate turnover and bioavailable phosphate concentrations exhibited pronounced seasonal variability characterized by extended periods of minima during summer months. The combination of rapid phosphate cycling (< 5 h), enhanced by elevated temperatures, and low concentrations of bioavailable phosphate (<11 nM) indicate the summer in the coastal Northwest Mediterranean is a period of phosphate limitation.

In the Northwest Mediterranean periods of phosphate limitation (*T*_*0*_ < 6 h) were associated with enhanced uptake and rapid cycling *d*-eDNA. Degrees of phosphate limitation (high, medium, low) were empirically determined for this coastal system based on the seasonal progression of phosphate turnover. The turnover of *d*-eDNA ranged from 3 h during high phosphate limitation to 33 days at low limitation. These results suggest that microbial utilization of *d*-eDNA, as a dissolved organic phosphorous (DOP) substrate [35,50], is an important factor regulating *d*-eDNA persistence in natural marine settings. However, there are numerous factors in the natural environment that could vary to result in the observed seasonal variance of *d*-eDNA persistence. DNA may be denatured directly by abiotic factors such as temperature, UV radiation, pH, dissolved oxygen and salinity [26,39–41,43]. However, some of these considerations are based on extreme environmental gradients (e.g. hypersaline conditions, anoxia, light vs darkness), and may be an inadequate replicate for the seasonally occurring environmental gradients that occur in natural marine systems. For example, in the present study pH ranged from 8.2–8.5, salinity from 36.6–38.2, and dissolved oxygen concentrations from 4.7–6.2 mL L^-1^, but nevertheless correspond to DNA persistence ranging from several hours to over a month.

In the context of biomonitoring in marine coastal waters, quantifying *d*-eDNA turnover and elucidating the factors responsible for its persistence under natural conditions is necessary. A partial least squares regression (PLSR) model was constructed in an attempt to predict microbial uptake of *d*-eDNA and resulting turnover from seasonal variation in environmental conditions. The PLSR model was capable of explaining 60% of the variance in *d*-eDNA turnover from the observed parameters. Biologically available phosphate concentrations and phosphate turnover, both taken as measures for the degree of phosphate limitation, were the most important factors for predicting *d*-eDNA turnover. Other important biotic factors were chlorophyll and the concentration of dissolved inorganic nitrogen, likely reflecting the onset of phosphate limitation following the spring bloom. The influence of abiotic factors was less important and may be due in part to the weak seasonal gradients of pH, oxygen, UV radiation and salinity occurring at 3 m in the Northwest Mediterranean in comparison to more extreme environmental transitions (e.g. hypersalinity and anoxia). In a natural coastal system, microbial dependence on DOP during periods of phosphate scarcity appears to exert an important control on the seasonal persistence of *d*-eDNA. In the statistical model, approximately 40% of the variance in the observed, microbially mediated, turnover of *d*-eDNA remains unexplained. The PLSR model does not incorporate any information on microbial community structure. The presence of certain bacterial taxa adapted to utilization of DNA as a phosphorus source could explain some of the variance in the model. Notably the largest discrepancy occurs during days 250–300, a period corresponding to the breakdown of summer stratification and shifts in microbial diversity in the Northwest Mediterranean [72]. Incorporating quantification of phylogenetic structure may further constrain the links between microbial nutrient dynamics and *d*-eDNA turnover.

Marine microbes possess a range of strategies for dealing with inorganic P-limitation that either reduces cellular P requirements [86,87] or enzymatically hydrolyses the dissolved organic phosphorous pool [87–91]. Activities of alkaline phosphatase (APA), the enzyme that hydrolyses P-ester bonds in ATP and DNA, are typically enhanced at low SRP concentrations [48,92] and taken as evidence of microbes switching to DOP utilization under P limitation. However, APA enzymatic activities are notably difficult to interpret quantitatively [88] and thus provide little information on rate processes. Direct measurements of ATP uptake using radioactive bioassay approaches are less common: bulk ATP uptake rates measured in P-limited oligotrophic open ocean environments range from 0.22 ± 0.1 nM d^-1^ in the North Atlantic Subtropical gyre [79] to 2.0 ± 0.8 nM d^-1^ in the North Pacific Subtropical gyre [91], although they have been reported as high as 309 nM d^-1^ in extremely P-limited (PO_4_^3-^ *T*_*0*_ = 0.4 h) mesocosm experiments [35]. In the present study, average ATP uptake was 8.9 ± 12 nM d-^1^, which although is broadly consistent with previous studies covers a much wider range (0.23–40.8 nM d-^1^) owing to the seasonal variation absent in previous observations.

ATP uptake is commonly used as a model compound to estimate dissolved organic phosphorous (DOP) utilization [89,91,92]. However, the present study is primarily concerned with seasonal variance in the utilization of *d*-eDNA. Earlier work has reported uptake of dissolved DNA in the range of 1.8–8.0 μg L^-1^ h^-1^ [29,35,93]. In the present study dissolved DNA uptake ranges from 0.001–4.5 μg L^-1^ h^-1^, which is comparable to previous estimates, but as is the case for ATP, covers a broader range indicative of significant seasonal variability. Although ATP and *d*-eDNA turnover follow the same seasonal patterns related to phosphorous limitation, turnover of *d*-eDNA is typically slower. There are very few studies directly comparing turnover of DNA and ATP, but mesocosm enrichment experiments also describe slower turnover rates for DNA [35,50]. The data presented here indicates that ATP and *d*DNA utilization by microbial communities may not be directly comparable, consistent with previous studies that suggest the hydrolysis of DNA involves more complex enzymatic processes than the degradation of monomeric DOP substrates [94].

The similar seasonal patterns observed in ATP and DNA uptake, in addition to leucine incorporation rates, suggest microbes use *d*-eDNA in response to phosphorous limitation. Previous studies have used cell sorting to demonstrate the utilization of ATP as a phosphorous substrate by individual microbial groups including photosynthetic cyanobacteria, heterotrophic bacteria and eukaryotic phytoplankton [87–91]. Considering the hydrolytic enzymes involved in cleaving the P-ester bond, it is reasonable to postulate that the same groups of microbes are responsible for the uptake of *d*-eDNA. Although no direct cell-specific incorporation rates for DNA exist, size-fractionated studies in an experimental mesocosm show DNA uptake to be concentrated in organisms <10 μm [35] and in limnic systems to be concentrated in the bacterial size fraction [29,94]. It is also possible that microbial community structure can influence *d*-eDNA kinetics, if certain phylogenetic groups are differentially limited by phosphate [95,96]. In support of this hypothesis, time-series observations of microbial community structure in the Northwest Mediterranean appear to suggest that seasonal patterns in SAR 11 ecotype abundance are partly linked to phosphorous dynamics [72]. However, in natural systems it is difficult to differentiate whether the distribution of species can drive phosphorous dynamics [97], and by extension *d*-eDNA persistence, or if microbial community structure is an emergent property of environmental filtering [71,72].

The methodology employed in the current study measures biological utilization of the dissolved fraction of the environmental DNA pool. It remains unclear to what extent these results could be generalized for the entire pool of eDNA. There are no studies addressing the seasonal persistence of different size fractions of eDNA in natural marine settings. However, one quantitative experimental study showed that the dissolved fraction (<0.2 μm) accounted for >50% of total eDNA and 7–25% of Carp eDNA [98], emphasizing the potential significance of the dissolved fraction. Furthermore, fish DNA has been observed at higher concentrations in precipitated versus filtered samples [24], implying both intra- and extracellular sources exist, although methodological differences between filtration and precipitation render such comparisons difficult. Nevertheless, the size distribution of eDNA within the dissolved fraction may also vary [99] and individual components of the dissolved pool can be cycled at different rates [34,100]. The quantitative partitioning of eDNA across different size fractions is likely to depend on the mechanism through which it is introduced into the environment [24]. Urea production from fish, for example, might be better represented in the dissolved fraction than sloughing of epithelial cells or fecal matter. In the latter case, membrane structures might offer some protection to microbial degradation [101,102] that may not be captured with the methodology employed in this study. More research is certainly required on the size distribution of eDNA in natural environments, and in particular how production and decay rates might vary between target organisms and study sites. For example, although the present study highlights the importance of phosphorous dynamics for *d*-eDNA persistence in the natural marine environment, it is important to recognize that P-limitation may be less significant in other environments such as eutrophic or deep-sea ecosystems.

## Conclusions

The present study addressed the seasonal variability of dissolved eDNA turnover in marine surface waters of the Northwest Mediterranean. Significant variation in the persistence of *d*-eDNA was linked to microbial utilization of DNA as an organic phosphorous substrate under conditions of seasonal phosphate limitation. The future application of eDNA as a long-term biomonitoring tool for biodiversity and conservation should take seasonal variability into account, particularly in phosphate-limited water bodies. It is hypothesized that seasonal variability in eDNA turnover exists in other aquatic settings, although it may be driven by factors other than phosphorous limitation. Furthermore, it is suggested that the seasonal persistence of eDNA is decoupled across naturally occurring size fractions, resulting in differential artifacts depending on the target organism(s) and the environment in which they occur. In order to correctly understand the spatial and temporal scales of e-DNA species detection, future studies should aim to constrain the natural variability in DNA degradation kinetics for the system in question.

## Supporting information

S1 File PLSR model validation. Description of the model assumptions and cross validation technique used in PLSR development.
(PDF)

S2 File Source code. Archive of R source code used in the manuscript.
(PDF)

S3 File Compressed raw data files. Files containing all of the raw data used in the manuscript.
(ZIP)
