# Supplementary material for: Seasonal variability in the persistence of dissolved environmental DNA (eDNA) in a marine system: The role of microbial nutrient limitation
Source: PLoS One. 2018 Feb 23;13(2):e0192409. doi: 10.1371/journal.pone.0192409 (PMC5825020; doi:10.1371/journal.pone.0192409)
Supplement: S1 File — Description of the model assumptions and cross validation technique used in PLSR development. (PDF) [file pone.0192409.s001.pdf]

Supplementary Information 1: Assumption and validation of PLS model.

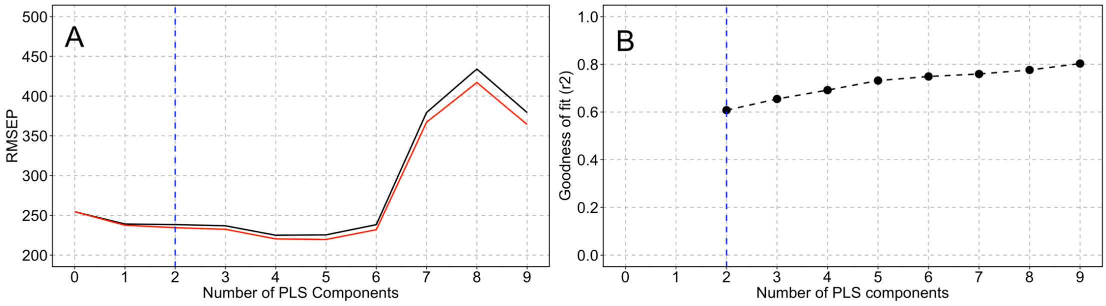

Fig S1: Validation of Partial Least Squares Regression Model. Panel A shows the root mean squared error of prediction (RMSEP) as a function of the number of components used in the PLS model. RMSEP was calculated from k-fold cross validation (k=10 segments) using the PLS R package [1], source code is provided below. The black line shows RMSEP for the cross-validated model and bias adjusted cross-validated model [1]. Panel B shows the goodness of fit between model prediction and observed data of DNA turnover as a function of the number of components used in PLS model. In both panels, the vertical blue dashed line denotes the number of PLS components used in the final version of the model.

Assumptions and validation

Partial least squares regression-modelling works by attempting to maximise the covariance in orthogonal space between x scores (T components) and y scores (U components), rather than the variables themselves. Consequently it does not suffer from the same assumptions concerning data structure as multiple linear regression modelling, and crucially is not invalidated by covariance in predictor variables. It is therefore well suited to environmental time-series data, which is typically characterised by high degrees of covariance amongst predictor variables. A theoretical assumption is that there is a tendency for covariance between T and U components of the PLS model, however, this is implicitly accounted for in its ability to predict the response variable, in this case DNA turnover (Fig. S1B).

An important assumption in model development is the number of PLS components selected to build the model. In theory the predictive capability of the model increases when more components are included (e.g. Fig. S1B). However, this introduces additional complexity that shifts the emphasis of the model away from describing the main features in the data to describing the noise (Fig. S1A). PLS models can suffer from over-fitting if the number of PLS components are not selected objectively. A commonly accepted method is to look at the root mean square error of prediction (RMSEP) when predicting new samples [2] (Fig. S1A). This can be achieved using cross-validation when building the model. Various methods of internal cross validation are available, including the leave one out (LOO) method and k-fold cross validation (CV). In the present PLS model, CV was chosen over LOO as it is typically considered a more robust form of cross validation [2]. In k-fold cross validation the data are divided into k equally sized segments, commonly referred to as folds. Subsequently k iterations of training and validation are performed so that for each iteration, one fold of data is removed for validation whilst the remaining *k-1*

folds are used for training the model [2]. The RMSEP is then the mean prediction error for each fold of data removed during cross validation. The numbers of components that give the lowest RMSEP are considered optimal for the model. 10-fold cross validation is most common and that is the approach used here to select the two component model. Furthermore, in some cases, RMSEP can increase with more components, as is the case in the present model where RMSEP is comparably low for 2-6 components and then increases from 7-9 components (Fig S1A). However, increasing the number of components did not significantly impact predictability of the model above two components (Fig. S1B). Based on these considerations, a two component PLS model was used in the present study.

The R package PLSdepot [3] was used to construct the final version of the model. To validate the analysis described above, the model was initially ran blind and allowed to select the number of components based on the results of cross validation. In this mode, the PLS model was also optimised as a two-component solution (see PLSdepot source code below). Standardized coefficients were extracted from the two-component model to examine the significance of environmental parameters.

A possible limitation with the present model is that the small dataset does not allow one to assess the predictability of the model for new samples. To do this one would need to remove a large segment of data to validate against a training model. However, since the dataset here covers one seasonal cycle, containing 18 observations, a sub-set training module would not accurately reflect the seasonality reflecting most of the variance in the data. However, whilst this may be considered a limitation from a pure PLS standpoint, the objective here was not to build a predictive PLS model for future unknown samples, as is sometimes the case in PLS model development. Rather the objective was to use PLS as a statistical model capable of predicting the variation in DNA turnover from measured environmental parameters from the present set of data. The internal cross-validation approach described above is adequate for this objective [2].

.

R Source code for PLS model construction

```
#PLS Package (Mevick et al. 2016)

PLS_DNA1 <- plsr(DNAturnover ~ Pturnover + Chl + DIN + PO4 + pH +
salinity + Temperature + Oxygen + Daylength, data=PLSDNAturnover_std,
ncomp=9, validation ="CV")

Summary (PLS_DNA1)

#plsdepot package (Sanchez, 2016)

PLSDNAturnover_pls0c <- plsreg1(PLSDNAturnover [,1:9], PLSDNAturnover
[,10,drop=FALSE], comps=NULL, crosval=TRUE)

#To inspect number of components optimized by cross validation
PLSDNAturnover_pls0c$y.scores

#To run model with two components

PLSDNAturnover_pls2c <- plsreg1(PLSDNAturnover [,1:9], PLSDNAturnover
[,10,drop=FALSE], comps=2, crosval=TRUE)
```

```
#To inspect standardized coefficents

PLSDNAturnover_pls2c$std.coefs

#End
```

References

1. Mevik B-H, Wehrens R. Introduction to the pls Package. 2016; 1–24.
2. Westerhuis JA, Hoefsloot HCJ, Smit S, Vis DJ, Smilde AK, Velzen EJJ, et al. Assessment of PLSDA cross validation. Metabolomics. 2008; doi:10.1007/s11306-007-0099-6
3. Sanchez G. Package "plsdepot" Title Partial Least Squares (PLS) Data Analysis Methods. 2016.
