# Supplementary material for: Seasonal variability in the persistence of dissolved environmental DNA (eDNA) in a marine system: The role of microbial nutrient limitation
Source: PLoS One. 2018 Feb 23;13(2):e0192409. doi: 10.1371/journal.pone.0192409 (PMC5825020; doi:10.1371/journal.pone.0192409)
Supplement: S2 File — Archive of R source code used in the manuscript. (PDF) [file pone.0192409.s002.pdf]

## S2 File: R source code

**#R Source code for producing Figure 1A**

```
guestturncomp1 <-ggplot(guestturncomp, aes(x=guestturnmeas,
y=guestturnbio)) + geom_point(size=4, shape=16, stroke=1) +
geom_smooth(method=lm)

guestturncomp2 <- guestturncomp1 + theme(panel.border =
element_rect(colour = "black", fill=NA, size=1))

guestturncomp3 <- guestturncomp2 + ylab("Turnover (h)") + xlab("Non-
labeled substrate addition (nM)")

guestturncomp4 <- guestturncomp3 + theme(axis.text.x =
element_text(size = 18, colour = "black"), axis.text.y =
element_text(size = 18, colour = "black"))

guestturncomp5 <- guestturncomp4 + ylim(0,40) + xlim(-10,50)

guestturncomp6 <- guestturncomp5 + scale_y_continuous(breaks=c(0, 10,
20, 30, 40)) + scale_x_continuous(breaks=c(-10, 0, 10, 20, 30, 40,
50)) + expand_limits(x=c(-10,50), y=c(0,40))

guestturncomp7 <- guestturncomp6 + theme(panel.background =
element_rect(fill="white", colour="black")) +
theme(panel.grid.major=element_line(colour="grey", linetype =
"dashed"))

guestturncomp8 <- guestturncomp7 + annotate("text", x = -10, y =
37.5, label = "A", size=12, fontface=1)

guestturncomp9 <- guestturncomp8 + theme(legend.position="none",
axis.title.x=element_text(size=18),axis.title.y=element_text(size=18)
)

guestturncomp9

guestturncomp10 <- guestturncomp9 + geom_segment(aes(x = -5, y = 0,
xend = 0, yend = 3.1, colour = "blue", linetype="dashed"))

guestturncomp11 <- guestturncomp10 + geom_vline(xintercept = 0)

guestturncomp11

tiff("Fig1A.tiff", width = 9, height = 5, units = 'in', res = 300)
plot(guestturncomp9) # Make plot
dev.off()


#Run linear model

guestturncomplm <- lm(formula = guestturnmeas~ guestturnbio, data =
guestturncomp)

#call linear model summary

summary(guestturncomplm)

Call:
lm(formula = guestturnmeas ~ guestturnbio, data = guestturncomp)
```

**(excluding 1200 hr time point)**

```
Residuals:
    Min      1Q  Median      3Q     Max 
-37.923   1.268   2.791   3.944   9.066 

Coefficients:
            Estimate Std. Error t value Pr(>|t|)    
(Intercept) -2.57042    3.43482  -0.748    0.466    
guestturnbio   0.91805    0.03406  26.952 4.03e-14 ***
---
Signif. codes:  
0 '***' 0.001 '**' 0.01 '*' 0.05 '.' 0.1 ' ' 1

Residual standard error: 10.86 on 15 degrees of freedom
Multiple R-squared:  0.9798,  Adjusted R-squared:  0.9784 
F-statistic: 726.4 on 1 and 15 DF,  p-value: 4.034e-14
```

**All data (including 1200 hr time point)**

```
lm(formula = guestturnmeas ~ guestturnbio, data = guestturncomp)

Residuals:
    Min      1Q  Median      3Q     Max 
-97.917  -3.772   3.613   6.397  82.869 

Coefficients:
            Estimate Std. Error t value Pr(>|t|)    
(Intercept) -6.00981   10.48613  -0.573    0.575    
guestturnbio   1.02280    0.03948  25.909 1.71e-14 ***
---
Signif. codes:  
0 '***' 0.001 '**' 0.01 '*' 0.05 '.' 0.1 ' ' 1

Residual standard error: 34.67 on 16 degrees of freedom
Multiple R-squared:  0.9767,  Adjusted R-squared:  0.9753 
F-statistic: 671.3 on 1 and 16 DF,  p-value: 1.712e-14
```

**#R Source code for producing Figure 1B**

```
pturncomp <-ggplot(pturncomp, aes(x=pturnmeas, y=pturnbio)) + 
geom_point(size=4, shape=16, stroke=1) + geom_smooth(method=lm) + 
geom_errorbarh(aes(xmin=pturnmeas-errorpturnmeas, 
xmax=pturnmeas+errorpturnmeas), height=3) + 
geom_errorbar(aes(ymin=pturnbio-errorpturnbio, 
ymax=pturnbio+errorpturnbio), width=3)

pturncomp1 <-ggplot(pturncomp, aes(x=pturnmeas, y=pturnbio)) + 
geom_point(size=4, shape=16, stroke=1) + geom_smooth(method=lm) + 
geom_errorbarh(aes(xmin=pturnmeas-errorpturnmeas, 
xmax=pturnmeas+errorpturnmeas), height=3) + 
geom_errorbar(aes(ymin=pturnbio-errorpturnbio, 
ymax=pturnbio+errorpturnbio), width=3)

pturncomp2 <- pturncomp1 + theme(panel.border = element_rect(colour = 
"black", fill=NA, size=1))

pturncomp3 <- pturncomp2 + ylab("Phosphate turnover Bioassay (h)") + 
xlab("Phosphate turnover (h)")
```

```
pturncomp4 <- pturncomp3 + theme(axis.text.x = element_text(size =
18, colour = "black"), axis.text.y = element_text(size = 18, colour =
"black"))

pturncomp5 <- pturncomp4 + ylim(0,150) + xlim(0,150)

pturncomp6 <- pturncomp5 + scale_y_continuous(breaks=c(0, 25, 50, 75,
100, 125, 150)) + scale_x_continuous(breaks=c(0, 25, 50, 75, 100,
125, 150)) + expand_limits(x=c(0,150), y=c(0,150))

pturncomp7 <- pturncomp6 + theme(panel.background =
element_rect(fill="white", colour="black")) +
theme(panel.grid.major=element_line(colour="grey", linetype =
"dashed"))

pturncomp8 <- pturncomp7 + annotate("text", x = 0, y = 150, label =
"B", size=12, fontface=1)

pturncomp9 <- pturncomp8 + theme(legend.position="none",
axis.title.x=element_text(size=18),axis.title.y=element_text(size=18)
)

pturncomp9

tiff("Fig1B.tiff", width = 9, height = 5, units = 'in', res = 300)
plot(pturncomp9) # Make plot
dev.off()


#Run linear model

pturncomplm <- lm(formula = pturnmeas~ pturnbio, data = pturncomp)

#call linear model summary

#summary(pturncomplm)

Residuals:
    Min      1Q  Median      3Q     Max
-6.0717 -0.2365 -0.1480  0.7934  8.1785

Coefficients:
            Estimate Std. Error t value Pr(>|t|)
(Intercept)  0.20278    0.99384   0.204    0.841
pturnbio     1.01382    0.02003  50.608   <2e-16 ***
---
Signif. codes:
0 '***' 0.001 '**' 0.01 '*' 0.05 '.' 0.1 ' ' 1

Residual standard error: 3.297 on 15 degrees of freedom
Multiple R-squared:  0.9942,	Adjusted R-squared:  0.9938
F-statistic:  2561 on 1 and 15 DF,  p-value: < 2.2e-16
```

**#R Source code for producing Figure 1C**

```
atpturncomp1 <-ggplot(atpturncomp, aes(x=atpturnmeas, y=atpturnbio))
+ geom_point(size=4, shape=16, stroke=1) + geom_smooth(method=lm) +
geom_errorbarh(aes(xmin=atpturnmeas-erroratpturnmeas,
xmax=atpturnmeas+erroratpturnmeas), height=3) +
```

```
geom_errorbar(aes(ymin=atpturnbio-erroratpturnbio,
ymax=atpturnbio+erroratpturnbio), width=3)

atpturncomp2 <- atpturncomp1 + theme(panel.border =
element_rect(colour = "black", fill=NA, size=1))

atpturncomp3 <- atpturncomp2 + ylab("ATP turnover Bioassay (h)") +
xlab("ATP turnover (h)")

atpturncomp4 <- atpturncomp3 + theme(axis.text.x = element_text(size
= 18, colour = "black"), axis.text.y = element_text(size = 18, colour
= "black"))

atpturncomp5 <- atpturncomp4 + ylim(0,150) + xlim(0,150)

atpturncomp6 <- atpturncomp5 + scale_y_continuous(breaks=c(0, 50,
100, 150, 200, 250, 300, 350)) + scale_x_continuous(breaks=c(0, 50,
100, 150, 200, 250, 300, 350)) + expand_limits(x=c(0,350),
y=c(0,350))

atpturncomp7 <- atpturncomp6 + theme(panel.background =
element_rect(fill="white", colour="black")) +
theme(panel.grid.major=element_line(colour="grey", linetype =
"dashed"))

atpturncomp8 <- atpturncomp7 + annotate("text", x = 0, y = 325, label
= "C", size=12, fontface=1)

atpturncomp9 <- atpturncomp8 + theme(legend.position="none",
axis.title.x=element_text(size=18),axis.title.y=element_text(size=18)
)

atpturncomp9

tiff("Fig1C.tiff", width = 9, height = 5, units = 'in', res = 300)
plot(atpturncomp9) # Make plot
dev.off()


#Run linear model

atpturncomplm <- lm(formula = atpturnmeas~ atpturnbio, data =
atpturncomp)

#call linear model summary

summary(atpturncomplm)

Call:
lm(formula = atpturnmeas ~ atpturnbio, data = atpturncomp)
```

**(excluding 1200 hr time point)**

```
Residuals:
    Min      1Q  Median      3Q     Max 
-37.923   1.268   2.791   3.944   9.066 

Coefficients:
            Estimate Std. Error t value Pr(>|t|)    
(Intercept) -2.57042    3.43482  -0.748    0.466
```

```
atpturnbio   0.91805    0.03406  26.952 4.03e-14 ***
---
Signif. codes:
0 '***' 0.001 '**' 0.01 '*' 0.05 '.' 0.1 ' ' 1

Residual standard error: 10.86 on 15 degrees of freedom
Multiple R-squared:  0.9798,  Adjusted R-squared:  0.9784
F-statistic: 726.4 on 1 and 15 DF,  p-value: 4.034e-14
```

**All data (including 1200 hr time point)**

```
Residuals:
    Min      1Q  Median      3Q     Max
-40.545   0.025   3.943   5.103   6.394

Coefficients:
             Estimate Std. Error t value Pr(>|t|)
(Intercept) -5.352156   2.907028  -1.841   0.0842 .
atpturnbio   0.964631   0.009267 104.094   <2e-16 ***
---
Signif. codes:
0 '***' 0.001 '**' 0.01 '*' 0.05 '.' 0.1 ' ' 1

Residual standard error: 11.2 on 16 degrees of freedom
Multiple R-squared:  0.9985,  Adjusted R-squared:  0.9984
F-statistic: 1.084e+04
```

**#R Source code for producing Figure 1D**

```
dnaturncomp1 <-ggplot(dnaturncomp, aes(x=dnaturnmeas, y=dnaturnbio))
+ geom_point(size=4, shape=16, stroke=1) + geom_smooth(method=lm) +
geom_errorbarh(aes(xmin=dnaturnmeas-errordnaturnmeas,
xmax=dnaturnmeas+errordnaturnmeas), height=3) +
geom_errorbar(aes(ymin=dnaturnbio-errordnaturnbio,
ymax=dnaturnbio+errordnaturnbio), width=3)

dnaturncomp2 <- dnaturncomp1 + theme(panel.border =
element_rect(colour = "black", fill=NA, size=1))

dnaturncomp3 <- dnaturncomp2 + ylab("DNA turnover Bioassay (h)") +
xlab("DNA turnover (h)")

dnaturncomp4 <- dnaturncomp3 + theme(axis.text.x = element_text(size
= 18, colour = "black"), axis.text.y = element_text(size = 18, colour
= "black"))

dnaturncomp5 <- dnaturncomp4 + ylim(0,600) + xlim(0,600)

dnaturncomp6 <- dnaturncomp5 + scale_y_continuous(breaks=c(0, 100,
200, 300, 400, 500, 600)) + scale_x_continuous(breaks=c(0, 100, 200,
300, 400, 500, 600)) + expand_limits(x=c(0,600), y=c(0,600))

dnaturncomp7 <- dnaturncomp6 + theme(panel.background =
element_rect(fill="white", colour="black")) +
theme(panel.grid.major=element_line(colour="grey", linetype =
"dashed"))

dnaturncomp8 <- dnaturncomp7 + annotate("text", x = 0, y = 550, label
= "D", size=12, fontface=1)
```

```
dnaturncomp9 <- dnaturncomp8 + theme(legend.position="none",
axis.title.x=element_text(size=18),axis.title.y=element_text(size=18)
)

dnaturncomp9

tiff("Fig1D.tiff", width = 9, height = 5, units = 'in', res = 300)
plot(dnaturncomp9) # Make plot
dev.off()


#Run linear model

dnaturncomplm <- lm(formula = dnaturnmeas~ dnaturnbio, data =
dnaturncomp)

#call linear model summary

summary(dnaturncomplm)

Call:
lm(formula = dnaturnmeas ~ dnaturnbio, data = dnaturncomp)
```

**(excluding 1200 hr time point)**

```
Residuals:
    Min      1Q  Median      3Q     Max
-37.923   1.268   2.791   3.944   9.066

Coefficients:
            Estimate Std. Error t value Pr(>|t|)
(Intercept) -2.57042    3.43482  -0.748    0.466
dnaturnbio   0.91805    0.03406  26.952 4.03e-14 ***
---
Signif. codes:
0 '***' 0.001 '**' 0.01 '*' 0.05 '.' 0.1 ' ' 1

Residual standard error: 10.86 on 15 degrees of freedom
Multiple R-squared:  0.9798,  Adjusted R-squared:  0.9784
F-statistic: 726.4 on 1 and 15 DF,  p-value: 4.034e-14
```

**All data (including 1200 hr time point)**

```
lm(formula = dnaturnmeas ~ dnaturnbio, data = dnaturncomp)

Residuals:
    Min      1Q  Median      3Q     Max
-97.917  -3.772   3.613   6.397  82.869

Coefficients:
            Estimate Std. Error t value Pr(>|t|)
(Intercept) -6.00981   10.48613  -0.573    0.575
dnaturnbio   1.02280    0.03948  25.909 1.71e-14 ***
---
Signif. codes:
0 '***' 0.001 '**' 0.01 '*' 0.05 '.' 0.1 ' ' 1

Residual standard error: 34.67 on 16 degrees of freedom
Multiple R-squared:  0.9767,  Adjusted R-squared:  0.9753
F-statistic: 671.3 on 1 and 16 DF,  p-value: 1.712e-14
```

```
#End
```

**#R code for Figure 3**

```
#Load packages ggplot and Rcolorbrewer

library("ggplot2",
lib.loc="/Library/Frameworks/R.framework/Versions/3.2/Resources/libra
ry")

library("RColorBrewer",
lib.loc="/Library/Frameworks/R.framework/Versions/3.2/Resources/libra
ry")

#Import csv files (contained as supplementary information
```

**#Figure 3A – Temperature (Fig3temp.csv)**

```
Fig3temp <- read_csv("~/Documents/Work/Publications/2017/p dynamics
and eDNA/Revised Manuscript/Revised Figures/Figure 3/Fig.
3/Fig3temp.csv")

View(Fig3temp)
```

**#Figure 3B – Salinity (Fig3sal.csv)**

```
Fig3sal <- read_csv("~/Documents/Work/Publications/2017/p dynamics
and eDNA/Revised Manuscript/Revised Figures/Figure 3/Fig.
3/Fig3sal.csv")

View(Fig3sal)
```

**#Figure 3C – Inorganic phosphate and biologically available phosphate (Fig3pho.csv & Fig3bap.csv)**

```
Fig3pho <- read_csv("~/Documents/Work/Publications/2017/p dynamics
and eDNA/Revised Manuscript/Revised Figures/Figure 3/Fig.
3/Fig3pho.csv")

View(Fig3pho)

Fig3bap <- read_csv("~/Documents/Work/Publications/2017/p dynamics
and eDNA/Revised Manuscript/Revised Figures/Figure 3/Fig.
3/Fig3bap.csv")

View(Fig3bap)
```

**#Figure 3D – Chloropyhll (Fig3chl.csv)**

```
Fig3chl <- read_csv("~/Documents/Work/Publications/2017/p dynamics
and eDNA/Revised Manuscript/Revised Figures/Figure 3/Fig.
3/Fig3chl.csv")

View(Fig3chl)
```

**#Figure 3E – Dissolved Inorganic Nitrogen (Nitrate + Nitrite) (Fig3din.csv)**

```
Fig3din <- read_csv("~/Documents/Work/Publications/2017/p dynamics
and eDNA/Revised Manuscript/Revised Figures/Figure 3/Fig.
3/Fig3din.csv")

View(Fig3din)
```

**#Figure 3F – Dissolved Oxygen (Fig3oxy.csv)**

```
Fig3oxy <- read_csv("~/Documents/Work/Publications/2017/p dynamics
and eDNA/Revised Manuscript/Revised Figures/Figure 3/Fig.
3/Fig3din.csv")

View(Fig3oxy)
```

**#Plot Figure 3A - Temperature**

```
myColors <- c("black", "darkgrey")

names(myColors) <- levels(Fig3temp$Sample)

colScale <- scale_colour_manual(name = "",values = myColors)

Fig3temp$Sample <- factor(Fig3temp$Sample)

Temperature <- ggplot(Fig3temp, aes(Yearday, Temperature, colour =
Sample)) + geom_point(size=4, shape = 16, stroke = 1) +
geom_line(colour="black", size =0.8, linetype="dashed")

Temperature1 <- Temperature + colScale

Temperature1

Temperature2 <- Temperature1 + theme(panel.border =
element_rect(colour = "black", fill=NA, size=1))

Temperature2

Temperature3 <- Temperature2 + theme(legend.position="none",
axis.title.x=element_text(size=18),axis.title.y=element_text(size=18)
)

Temperature3

Temperature4 <- Temperature3 +
ylab(expression("Temperature"*~~degree*C))

Temperature4

Temperature5 <- Temperature4 + xlab("Day")

Temperature5

Temperature6 <- Temperature5 + theme(axis.text.x = element_text(size
= 18, colour = "black"), axis.text.y = element_text(size = 18, colour
= "black"))

Temperature6

Temperature7 <- Temperature6 + ylim(0,30) + xlim(0,365)
```

```
Temperature7

Temperature8 <- Temperature7 + scale_y_continuous(breaks=c(0, 5, 10,
15, 20, 25)) +
scale_x_continuous(breaks=c(0,50,100,150,200,250,300,350)) +
expand_limits(x=c(0,365), y=c(0, 25))

Temperature8

Temperature9 <- Temperature8 + theme(panel.background =
element_rect(fill="white", colour="black")) +
theme(panel.grid.major=element_line(colour="grey", linetype =
"dashed"))

Temperature9

Temperature10 <- Temperature9 + annotate("text", x = 0, y = 22.5,
label = "A", size=12, fontface=1)

Temperature10

tiff("Fig3A.tiff", width = 9, height = 5, units = 'in', res = 300)
plot(Temperature10) # Make plot
dev.off()
```

**#Plot Figure 3B - Salinity**

```
myColors <- c("black", "darkgrey")

names(myColors) <- levels(Fig3sal$Sample)

colScale <- scale_colour_manual(name = "",values = myColors)

Fig3sal$Sample <- factor(Fig3sal$Sample)

Salinity <- ggplot(Fig3sal, aes(Yearday, Salinity, colour = Sample))
+ geom_point(size=4, shape = 16, stroke = 1) +
geom_line(colour="black", size =0.8, linetype="dashed")

Salinity1 <- Salinity + colScale

Salinity1

Salinity2 <- Salinity1 + theme(panel.border = element_rect(colour =
"black", fill=NA, size=1))

Salinity2

Salinity3 <- Salinity2 + theme(legend.position="none",
axis.title.x=element_text(size=18),axis.title.y=element_text(size=18)
)

Salinity3

Salinity4 <- Salinity3 + xlab("Salinity (PSU)")
Salinity4

Salinity5 <- Salinity4 + xlab("Day")

Salinity5
```

```
Salinity6 <- Salinity5 + theme(axis.text.x = element_text(size = 18,
colour = "black"), axis.text.y = element_text(size = 18, colour =
"black"))

Salinity6

Salinity7 <- Salinity6 + ylim(35,39) + xlim(0,365)

Salinity7

Salinity8 <- Salinity7 + scale_y_continuous(breaks=c(35, 36, 37, 38,
39)) + scale_x_continuous(breaks=c(0,50,100,150,200,250,300,350)) +
expand_limits(x=c(0,365), y=c(35, 39))

Salinity8

Salinity9 <- Salinity8 + theme(panel.background =
element_rect(fill="white", colour="black")) +
theme(panel.grid.major=element_line(colour="grey", linetype =
"dashed"))

Salinity9

Salinity10 <- Salinity9 + annotate("text", x = 0, y = 38.75, label =
"B", size=12, fontface=1)

Salinity10

tiff("Fig3B.tiff", width = 9, height = 5, units = 'in', res = 300)
plot(Salinity10) # Make plot
dev.off()
```

**#Plot Figure 3C – Inorganic phosphate and biologically available phosphate**

```
myColors <- c("black", "darkgrey")

names(myColors) <- levels(Fig3pho$Sample)

colScale <- scale_colour_manual(name = "",values = myColors)

Fig3pho$Sample <- factor(Fig3pho$Sample)

Phosphate <- ggplot(Fig3pho, aes(Yearday, Phosphate, colour =
Sample)) + geom_point(size=4, shape = 16, stroke = 1) +
geom_line(colour="black", size =0.8, linetype="dashed") +
geom_line(data = Fig3bap, aes(x = Yearday, y = BAP), color = "blue",
size=0.8)

Phosphate

Phosphate1 <- Phosphate + colScale

Phosphate1


Phosphate2 <- Phosphate1 + theme(panel.border = element_rect(colour =
"black", fill=NA, size=1))
```

```
Phosphate3 <- Phosphate2 + theme(legend.position = c(0.6,0.9)) +
theme(legend.text = element_text(size = 16)) + theme
(axis.title.x=element_text(size=18),axis.title.y=element_text(size=18
))

Phosphate3


Phosphate4 <- Phosphate3 + labs(y=expression(Phosphate~" "~nmol~L^{-
1}))

Phosphate4

Phosphate5 <- Phosphate4 + xlab("Day")

Phosphate5

Phosphate6 <- Phosphate5 + theme(axis.text.x = element_text(size =
18, colour = "black"), axis.text.y = element_text(size = 18, colour =
"black"))

Phosphate6

Phosphate7 <- Phosphate6 + ylim(0,80) + xlim(0,365)

Phosphate7

Phosphate8 <- Phosphate7 + scale_y_continuous(breaks=c(0, 20, 40, 60,
80)) + scale_x_continuous(breaks=c(0,50,100,150,200,250,300,350)) +
expand_limits(x=c(0,365), y=c(0, 80))

Phosphate8

Phosphate9 <- Phosphate8 + theme(panel.background =
element_rect(fill="white", colour="black")) +
theme(panel.grid.major=element_line(colour="grey", linetype =
"dashed"))

Phosphate9

Phosphate10 <- Phosphate9 + annotate("text", x = 0, y = 75, label =
"C", size=12, fontface=1) + annotate("text", x = 225, y = 66, label =
"k + Sn", size=6, color="blue", fontface=1)

Phosphate10

tiff("Fig3C.tiff", width = 9, height = 5, units = 'in', res = 300)
plot(Phosphate10) # Make plot
dev.off()


#Plot Figure 3D - Chlorophyll

myColors <- c("black", "darkgrey")

names(myColors) <- levels(Fig3chl$Sample)

colScale <- scale_colour_manual(name = "",values = myColors)

Fig3sal$Sample <- factor(Fig3chl$Sample)
```

```
Chlorophyll <- ggplot(Fig3chl, aes(Yearday, Chlorophyll, colour =
Sample)) + geom_point(size=4, shape = 16, stroke = 1) +
geom_line(colour="black", size =0.8, linetype="dashed")

Chlorophyll1 <- Chlorophyll + colScale

Chlorophyll1

Chlorophyll2 <- Chlorophyll1 + theme(panel.border =
element_rect(colour = "black", fill=NA, size=1))

Chlorophyll2

Chlorophyll3 <- Chlorophyll2 + theme(legend.position="none",
axis.title.x=element_text(size=18),axis.title.y=element_text(size=18)
)

Chlorophyll3

Chlorophyll4 <- Chlorophyll3 + labs(y=expression(Chlorophyll~"
"~mu~g~L^{-1}))

Chlorophyll4

Chlorophyll5 <- Chlorophyll4 + xlab("Day")

Chlorophyll5

Chlorophyll6 <- Chlorophyll5 + theme(axis.text.x = element_text(size
= 18, colour = "black"), axis.text.y = element_text(size = 18, colour
= "black"))

Chlorophyll6

Chlorophyll7 <- Chlorophyll6 + ylim(0,2) + xlim(0,365)

Chlorophyll7

Chlorophyll8 <- Chlorophyll7 + scale_y_continuous(breaks=c(0, 0.5, 1,
1.5, 2)) + scale_x_continuous(breaks=c(0,50,100,150,200,250,300,350))
+ expand_limits(x=c(0,365), y=c(0,2))

Chlorophyll8

Chlorophyll9 <- Chlorophyll8 + theme(panel.background =
element_rect(fill="white", colour="black")) +
theme(panel.grid.major=element_line(colour="grey", linetype =
"dashed"))

Chlorophyll9

Chlorophyll10 <- Chlorophyll9 + annotate("text", x = 0, y = 1.875,
label = "D", size=12, fontface=1)

Chlorophyll10

tiff("Fig3D.tiff", width = 9, height = 5, units = 'in', res = 300)
plot(Chlorophyll10) # Make plot
dev.off()
```

```
#Plot Figure 3E – Nitrate and Nitrite (Dissolved inorganic nitrogen)

myColors <- c("black", "darkgrey")

names(myColors) <- levels(Fig3din$Sample)

colScale <- scale_colour_manual(name = "",values = myColors)

Fig3sal$Sample <- factor(Fig3din$Sample)

Din <- ggplot(Fig3din, aes(Yearday, Din, colour = Sample)) +
geom_point(size=4, shape = 16, stroke = 1) +
geom_line(colour="black", size =0.8, linetype="dashed")

Din1 <- Din + colScale

Din1

Din2 <- Din1 + theme(panel.border = element_rect(colour = "black",
fill=NA, size=1))

Din2

Din3 <- Din2 + theme(legend.position="none",
axis.title.x=element_text(size=18),axis.title.y=element_text(size=18)
)

Din3

Din4 <- Din3 + labs(y=expression(Nitrate + Nitrite~" "~mu~mol~L^{-
1}))

Din4

Din5 <- Din4 + xlab("Day")

Din5

Din6 <- Din5 + theme(axis.text.x = element_text(size = 18, colour =
"black"), axis.text.y = element_text(size = 18, colour = "black"))

Din6

Din7 <- Din6 + ylim(35,39) + xlim(0,365)

Din7

Din8 <- Din7 + scale_y_continuous(breaks=c(0, 0.5, 1, 1.5, 2, 2.5,
3)) + scale_x_continuous(breaks=c(0,50,100,150,200,250,300,350)) +
expand_limits(x=c(0,365), y=c(0, 3))

Din8

Din9 <- Din8 + theme(panel.background = element_rect(fill="white",
colour="black")) + theme(panel.grid.major=element_line(colour="grey",
linetype = "dashed"))

Din9

Din10 <- Din9 + annotate("text", x = 0, y = 2.75, label = "E",
size=12, fontface=1)
```

```
Din10

tiff("Fig3E.tiff", width = 9, height = 5, units = 'in', res = 300)
plot(Din10) # Make plot
dev.off()
```

**#Plot Figure 3F – Dissolved oxygen**

```
myColors <- c("black", "darkgrey")

names(myColors) <- levels(Fig3oxy$Sample)

colScale <- scale_colour_manual(name = "",values = myColors)

Fig3oxy$Sample <- factor(Fig3oxy$Sample)

Oxygen <- ggplot(Fig3oxy, aes(Yearday, Oxygen, colour = Sample)) +
geom_point(size=4, shape = 16, stroke = 1) +
geom_line(colour="black", size =0.8, linetype="dashed")

Oxygen1 <- Oxygen + colScale

Oxygen1

Oxygen2 <- Oxygen1 + theme(panel.border = element_rect(colour =
"black", fill=NA, size=1))

Oxygen2

Oxygen3 <- Oxygen2 + theme(legend.position="none",
axis.title.x=element_text(size=18),axis.title.y=element_text(size=18)
)

Oxygen3

Oxygen4 <- Oxygen3 + labs(y=expression(Oxygen~" "~mg~L^{-1}))

Oxygen4

Oxygen5 <- Oxygen4 + xlab("Day")

Oxygen5

Oxygen6 <- Oxygen5 + theme(axis.text.x = element_text(size = 18,
colour = "black"), axis.text.y = element_text(size = 18, colour =
"black"))

Oxygen6

Oxygen7 <- Oxygen6 + ylim(4,7) + xlim(0,365)

Oxygen7

Oxygen8 <- Oxygen7 + scale_y_continuous(breaks=c(4, 4.5, 5, 5.5, 6,
6.5, 7)) + scale_x_continuous(breaks=c(0,50,100,150,200,250,300,350))
+ expand_limits(x=c(0,365), y=c(4,7))

Oxygen8
```

```
Oxygen9 <- Oxygen8 + theme(panel.background =
element_rect(fill="white", colour="black")) +
theme(panel.grid.major=element_line(colour="grey", linetype =
"dashed"))

Oxygen9

Oxygen10 <- Oxygen9 + annotate("text", x = 0, y = 6.75, label = "F",
size=12, fontface=1)

Oxygen10

tiff("Fig3F.tiff", width = 9, height = 5, units = 'in', res = 300)
plot(Oxygen10) # Make plot
dev.off()
```

**#R source code for Figure 4**

**#Fig. 4A – Phosphate turnover**

```
Photurn <- ggplot(Fig4Photurn, aes(Date, Photurn)) +
geom_errorbar(aes(ymin=Photurn-se, ymax=Photurn+se), width=3) +
geom_line(colour="black", size =0.8, linetype="dashed")+
geom_point(size=4, shape = 16, stroke = 1)

Photurn

Photurn1 <- Photurn

Photurn1

Photurn2 <- Photurn1 + theme(panel.border = element_rect(colour =
"black", fill=NA, size=1))

Photurn2

Photurn3 <- Photurn2 + theme(legend.position="none",
axis.title.x=element_text(size=18),axis.title.y=element_text(size=18)
)

Photurn3

Photurn4 <- Photurn3 + ylab("Phosphate turnover (h)")
Photurn4

Photurn5 <- Photurn4 + xlab("Day")

Photurn5

Photurn6 <- Photurn5 + theme(axis.text.x = element_text(size = 18,
colour = "black"), axis.text.y = element_text(size = 18, colour =
"black"))

Photurn6

Photurn7 <- Photurn6 + ylim(0,175) + xlim(0,365)

Photurn7
```

```
Photurn8 <- Photurn7 + scale_y_continuous(breaks=c(0, 25, 50, 75,
100, 125, 150, 175)) +
scale_x_continuous(breaks=c(0,50,100,150,200,250,300,350)) +
expand_limits(x=c(0,365), y=c(0,175))

Photurn8

Photurn9 <- Photurn8 + theme(panel.background =
element_rect(fill="white", colour="black")) +
theme(panel.grid.major=element_line(colour="grey", linetype =
"dashed"))

Photurn9

Photurn10 <- Photurn9 + annotate("text", x = 0, y = 162.5, label =
"A", size=12, fontface=1)

Photurn10

tiff("Fig4A.tiff", width = 9, height = 5, units = 'in', res = 300)
plot(Photurn10) # Make plot
dev.off()
```

**#Fig. 4B – Bacterial Production**

```
Bacpro <- ggplot(Fig4Bacpro, aes(Date, Bacpro)) +
geom_errorbar(aes(ymin=Bacpro-se, ymax=Bacpro+se), width=3) +
geom_line(colour="black", size =0.8, linetype="dashed")+
geom_point(size=4, shape = 16, stroke = 1)

Bacpro

Bacpro1 <- Bacpro

Bacpro1

Bacpro2 <- Bacpro1 + theme(panel.border = element_rect(colour =
"black", fill=NA, size=1))

Bacpro2

Bacpro3 <- Bacpro2 + theme(legend.position="none",
axis.title.x=element_text(size=18),axis.title.y=element_text(size=18)
)

Bacpro3

Bacpro4 <- Bacpro3 +
labs(y=expression(Bacterial~production~~(pmol~Leu~L^{-1}~h^{-1})))

Bacpro4

Bacpro5 <- Bacpro4 + xlab("Day")

Bacpro5

Bacpro6 <- Bacpro5 + theme(axis.text.x = element_text(size = 18,
colour = "black"), axis.text.y = element_text(size = 18, colour =
"black"))

Bacpro6
```

```
Bacpro7 <- Bacpro6 + ylim(0,80) + xlim(0,365)

Bacpro7

Bacpro8 <- Bacpro7 + scale_y_continuous(breaks=c(0, 20, 40, 60, 80))
+ scale_x_continuous(breaks=c(0,50,100,150,200,250,300,350)) +
expand_limits(x=c(0,365), y=c(0,80))

Bacpro8

Bacpro9 <- Bacpro8 + theme(panel.background =
element_rect(fill="white", colour="black")) +
theme(panel.grid.major=element_line(colour="grey", linetype =
"dashed"))

Bacpro9

Bacpro10 <- Bacpro9 + annotate("text", x = 0, y = 75, label = "B",
size=12, fontface=1)

Bacpro10

tiff("Fig4B.tiff", width = 9, height = 5, units = 'in', res = 300)
plot(Bacpro10) # Make plot
dev.off()
```

**#Fig. 4C – ATP turnover**

```
Atpturn <- ggplot(Fig4Atpturn, aes(Date, Atpturn)) +
geom_errorbar(aes(ymin=Atpturn-se, ymax=Atpturn+se), width=3) +
geom_line(colour="black", size =0.8, linetype="dashed")+
geom_point(size=4, shape = 16, stroke = 1)

Atpturn

Atpturn1 <- Atpturn

Atpturn1

Atpturn2 <- Atpturn1 + theme(panel.border = element_rect(colour =
"black", fill=NA, size=1))

Atpturn2

Atpturn3 <- Atpturn2 + theme(legend.position="none",
axis.title.x=element_text(size=18),axis.title.y=element_text(size=18)
)

Atpturn3

Atpturn4 <- Atpturn3 + ylab("ATP turnover (h)")
Atpturn4

Atpturn5 <- Atpturn4 + xlab("Day")

Atpturn5

Atpturn6 <- Atpturn5 + theme(axis.text.x = element_text(size = 18,
colour = "black"), axis.text.y = element_text(size = 18, colour =
"black"))
```

```
Atpturn6

Atpturn7 <- Atpturn6 + ylim(0,400) + xlim(0,365)

Atpturn7

Atpturn8 <- Atpturn7 + scale_y_continuous(breaks=c(0, 100, 200, 300,
400)) + scale_x_continuous(breaks=c(0,50,100,150,200,250,300,350)) +
expand_limits(x=c(0,365), y=c(0,400))

Atpturn8

Atpturn9 <- Atpturn8 + theme(panel.background =
element_rect(fill="white", colour="black")) +
theme(panel.grid.major=element_line(colour="grey", linetype =
"dashed"))

Atpturn9

Atpturn10 <- Atpturn9 + annotate("text", x = 0, y = 375, label = "C",
size=12, fontface=1)

Atpturn10

tiff("Fig4C.tiff", width = 9, height = 5, units = 'in', res = 300)
plot(Atpturn10) # Make plot
dev.off()
```

**#Fig. 4D – ATP uptake**

```
Atpupt <- ggplot(Fig4Atpupt, aes(Date, Atpupt)) +
geom_errorbar(aes(ymin=Atpupt-se, ymax=Atpupt+se), width=3) +
geom_line(colour="black", size =0.8, linetype="dashed")+
geom_point(size=4, shape = 16, stroke = 1)

Atpupt

Atpupt1 <- Atpupt

Atpupt1

Atpupt2 <- Atpupt1 + theme(panel.border = element_rect(colour =
"black", fill=NA, size=1))

Atpupt2

Atpupt3 <- Atpupt2 + theme(legend.position="none",
axis.title.x=element_text(size=18),axis.title.y=element_text(size=18)
)

Atpupt3

Atpupt4 <- Atpupt3 + labs(y=expression(ATP~uptake~~(nmol~L^{-1}~h^{-
1})))

Atpupt4

Atpupt5 <- Atpupt4 + xlab("Day")

Atpupt5
```

```
Atpupt6 <- Atpupt5 + theme(axis.text.x = element_text(size = 18,
colour = "black"), axis.text.y = element_text(size = 18, colour =
"black"))

Atpupt6

Atpupt7 <- Atpupt6 + ylim(0,2) + xlim(0,365)

Atpupt7

Atpupt8 <- Atpupt7 + scale_y_continuous(breaks=c(0, 0.5, 1, 1.5, 2))
+ scale_x_continuous(breaks=c(0,50,100,150,200,250,300,350)) +
expand_limits(x=c(0,365), y=c(0,2))

Atpupt8

Atpupt9 <- Atpupt8 + theme(panel.background =
element_rect(fill="white", colour="black")) +
theme(panel.grid.major=element_line(colour="grey", linetype =
"dashed"))

Atpupt9

Atpupt10 <- Atpupt9 + annotate("text", x = 0, y = 1.825, label = "D",
size=12, fontface=1)

Atpupt10

tiff("Fig4D.tiff", width = 9, height = 5, units = 'in', res = 300)
plot(Atpupt10) # Make plot
dev.off()
```

**#Fig. 4E – DNA turnover**

```
Dnaturn <- ggplot(Fig4Dnaturn, aes(Date, Dnaturn)) +
geom_errorbar(aes(ymin=Dnaturn-se, ymax=Dnaturn+se), width=3) +
geom_line(colour="black", size =0.8, linetype="dashed")+
geom_point(size=4, shape = 16, stroke = 1)

Dnaturn

Dnaturn1 <- Dnaturn

Dnaturn1

Dnaturn2 <- Dnaturn1 + theme(panel.border = element_rect(colour =
"black", fill=NA, size=1))

Dnaturn2

Dnaturn3 <- Dnaturn2 + theme(legend.position="none",
axis.title.x=element_text(size=18),axis.title.y=element_text(size=18)
)

Dnaturn3

Dnaturn4 <- Dnaturn3 + ylab("DNA turnover (d)")
Dnaturn4

Dnaturn5 <- Dnaturn4 + xlab("Day")
```

```
Dnaturn5

Dnaturn6 <- Dnaturn5 + theme(axis.text.x = element_text(size = 18,
colour = "black"), axis.text.y = element_text(size = 18, colour =
"black"))

Dnaturn6

Dnaturn7 <- Dnaturn6 + ylim(0,50) + xlim(0,365)

Dnaturn7

Dnaturn8 <- Dnaturn7 + scale_y_continuous(breaks=c(0, 10, 20, 30, 40,
50)) + scale_x_continuous(breaks=c(0,50,100,150,200,250,300,350)) +
expand_limits(x=c(0,365), y=c(0,50))

Dnaturn8

Dnaturn9 <- Dnaturn8 + theme(panel.background =
element_rect(fill="white", colour="black")) +
theme(panel.grid.major=element_line(colour="grey", linetype =
"dashed"))

Dnaturn9

Dnaturn10 <- Dnaturn9 + annotate("text", x = 0, y = 47.5, label =
"E", size=12, fontface=1)

Dnaturn10

tiff("Fig4E.tiff", width = 9, height = 5, units = 'in', res = 300)
plot(Dnaturn10) # Make plot
dev.off()
```

**#Fig. 4F – DNA uptake**

```
Dnaupt <- ggplot(Fig4Dnaupt, aes(Date, Dnaupt)) +
geom_errorbar(aes(ymin=Dnaupt-se, ymax=Dnaupt+se), width=3) +
geom_line(colour="black", size =0.8, linetype="dashed")+
geom_point(size=4, shape = 16, stroke = 1)

Dnaupt

Dnaupt1 <- Dnaupt

Dnaupt1

Dnaupt2 <- Dnaupt1 + theme(panel.border = element_rect(colour =
"black", fill=NA, size=1))

Dnaupt2

Dnaupt3 <- Dnaupt2 + theme(legend.position="none",
axis.title.x=element_text(size=18),axis.title.y=element_text(size=18)
)

Dnaupt3
```

```
Dnaupt4 <- Dnaupt3 + labs(y=expression(DNA~uptake~~(~mu~g~L^{-1}~h^{-
1})))

Dnaupt4

Dnaupt5 <- Dnaupt4 + xlab("Day")

Dnaupt5

Dnaupt6 <- Dnaupt5 + theme(axis.text.x = element_text(size = 18,
colour = "black"), axis.text.y = element_text(size = 18, colour =
"black"))

Dnaupt6

Dnaupt7 <- Dnaupt6 + ylim(0,1) + xlim(0,365)

Dnaupt7

Dnaupt8 <- Dnaupt7 + scale_y_continuous(breaks=c(0, 0.2, 0.4, 0.6,
0.8, 1)) + scale_x_continuous(breaks=c(0,50,100,150,200,250,300,350))
+ expand_limits(x=c(0,365), y=c(0,1))

Dnaupt8

Dnaupt9 <- Dnaupt8 + theme(panel.background =
element_rect(fill="white", colour="black")) +
theme(panel.grid.major=element_line(colour="grey", linetype =
"dashed"))

Dnaupt9

Dnaupt10 <- Dnaupt9 + annotate("text", x = 0, y = 0.9, label = "F",
size=12, fontface=1)

Dnaupt10

tiff("Fig4F.tiff", width = 9, height = 5, units = 'in', res = 300)
plot(Dnaupt10) # Make plot
dev.off()

#End
```

**#R Source code for Figure 5**

**#Fig. 5A**

```
myColors <- c("orangered", "lightgreen", "tan1")

names(myColors) <- levels(Phosphate_Turnover$Legend)

colScale <- scale_colour_manual(name = "",values = myColors)

Phosphate_Turnover$Legend <- factor(Phosphate_Turnover$Legend)

P_Turnover <- ggplot(Phosphate_Turnover, aes(Observation, Pt, colour
= Legend)) + geom_point(size=4, shape = 15, stroke = 1) +
geom_line(colour="black", size =0.8, linetype="dashed")

P_Turnover1 <- P_Turnover + colScale
```

```
P_Turnover1

P_Turnover2 <- P_Turnover1 + theme(panel.border = element_rect(colour
= "black", fill=NA, size=1))

P_Turnover2

P_Turnover3 <- P_Turnover2 + theme(legend.title=element_blank(),
axis.title.x=element_text(size=18),axis.title.y=element_text(size=18)
)

P_Turnover3

P_Turnover4 <- P_Turnover3 + ylab("Phosphate turnover (h)")

P_Turnover4

P_Turnover5 <- P_Turnover3 + xlab("Observation")

P_Turnover5

P_Turnover6 <- P_Turnover5 + theme(axis.text.x = element_text(size =
18, colour = "black"), axis.text.y = element_text(size = 18, colour =
"black"))

P_Turnover6

P_Turnover7 <- P_Turnover6 + ylim(0,150) + xlim(0,18)

P_Turnover7

P_Turnover8 <- P_Turnover7 + scale_y_continuous(breaks=c(0, 25, 50,
75, 100, 125, 150)) +
scale_x_continuous(breaks=c(0,1,2,3,4,5,6,7,8,9,10,11,12,13,14,15,16,
17,18,19,20)) + expand_limits(x=c(0,18), y=c(0, 150))


P_Turnover8

P_Turnover9 <- P_Turnover8 + theme(panel.background =
element_rect(fill="white", colour="black"))

P_Turnover9

P_Turnover10 <- P_Turnover9 + theme(legend.position=c(0.9, 0.85))

P_Turnover11 <- P_Turnover10 +
theme(panel.grid.major=element_line(colour="grey", linetype =
"dashed"))


P_Turnover12 <- P_Turnover11 + theme(legend.title=element_blank())


P_Turnover13 <- P_Turnover12 + theme(legend.text = element_text(size
= 16))

P_Turnover14 <- P_Turnover13 + ylab("Phosphate turnover (h)")

P_Turnover14
```

```
P_Turnover15 <- P_Turnover14 + annotate("text", x = 0, y = 137.5,
label = "A", size=12, fontface=1)

P_Turnover15

tiff("Fig5A.tiff", width = 9, height = 5, units = 'in', res = 300)
plot(P_Turnover15) # Make plot
dev.off()


#Fig. 5B

xorder <- c("Low", "Medium", "High")

Leu_Uptake_box_plot <-ggplot(Leu_Uptake, aes(Category, Value))

Leu_Uptake_box_plot


Leu_Uptake_box_plotv2 <- Leu_Uptake_box_plot + geom_boxplot(aes(fill
= Legend), outlier.colour = NA, notch=FALSE)

Leu_Uptake_box_plotv2


Leu_Uptake_box_plotv3 <- Leu_Uptake_box_plotv2 +
scale_x_discrete(limits=xorder)

Leu_Uptake_box_plotv3

Leu_Uptake_box_plotv4 <- Leu_Uptake_box_plotv3 +
scale_fill_manual(values=c("orangered", "lightgreen", "tan1" ))

Leu_Uptake_box_plotv4

Leu_Uptake_box_plotv5 <- Leu_Uptake_box_plotv4 + theme(panel.border =
element_rect(colour = "black", fill=NA, size=1))

Leu_Uptake_box_plotv5


Leu_Uptake_box_plotv6 <- Leu_Uptake_box_plotv5 +
theme(legend.title=element_blank(), axis.title.x =
element_text(size=18),axis.title.y=element_text(size=18))

Leu_Uptake_box_plotv6

Leu_Uptake_box_plotv7 <- Leu_Uptake_box_plotv6 +
labs(y=expression(Bacterial~production~~(pmol~Leu~L^{-1}~h^{-1})))

Leu_Uptake_box_plotv7

Leu_Uptake_box_plotv8 <- Leu_Uptake_box_plotv7 + xlab("Phosphate
Limitation")

Leu_Uptake_box_plotv8


Leu_Uptake_box_plotv9 <- Leu_Uptake_box_plotv8 +
theme(legend.position="none")
```

```
Leu_Uptake_box_plotv9

#Axis Options

Leu_Uptake_box_plotv10 <- Leu_Uptake_box_plotv9 + theme(axis.text.x =
element_text(size = 18, colour = "black"), axis.text.y =
element_text(size = 18, colour = "black"))

Leu_Uptake_box_plotv10

Leu_Uptake_box_plotv11 <- Leu_Uptake_box_plotv10 +
theme(panel.background = element_rect(fill="white", colour="black"))
+ theme(panel.grid.major=element_line(colour="grey", linetype =
"dashed")) + theme(legend.title=element_blank())

Leu_Uptake_box_plotv11

Leu_Uptake_box_plotv12 <- Leu_Uptake_box_plotv11 + annotate("text", x
= 0.6, y = 65, label = "B", size=12, fontface=1)

tiff("Fig5B.tiff", width = 9, height = 5, units = 'in', res = 300)
plot(Leu_Uptake_box_plotv12) # Make plot
dev.off()
```

**#Fig. 5C**

```
xorder <- c("Low", "Medium", "High")

ATP_turnover_box_plot <-ggplot(ATP_Turnover, aes(Category, Value))

ATP_turnover_box_plot


ATP_turnover_box_plotv2 <- ATP_turnover_box_plot +
geom_boxplot(aes(fill = Legend), outlier.colour = NA, notch=TRUE)

ATP_turnover_box_plotv2


ATP_turnover_box_plotv3 <- ATP_turnover_box_plotv2 +
scale_x_discrete(limits=xorder)

ATP_turnover_box_plotv3

ATP_turnover_box_plotv4 <- ATP_turnover_box_plotv3 +
scale_fill_manual(values=c("orangered", "lightgreen", "tan1" ))

ATP_turnover_box_plotv4

ATP_turnover_box_plotv5 <- ATP_turnover_box_plotv4 +
theme(panel.border = element_rect(colour = "black", fill=NA, size=1))

ATP_turnover_box_plotv5


ATP_turnover_box_plotv6 <- ATP_turnover_box_plotv5 +
theme(legend.title=element_blank(), axis.title.x =
element_text(size=18),axis.title.y=element_text(size=18))

ATP_turnover_box_plotv6
```

```
ATP_turnover_box_plotv7 <- ATP_turnover_box_plotv6 + ylab("ATP
turnover (h)")

ATP_turnover_box_plotv7

ATP_turnover_box_plotv8 <- ATP_turnover_box_plotv7 + xlab("Phosphate
Limitation")

ATP_turnover_box_plotv8


ATP_turnover_box_plotv9 <- ATP_turnover_box_plotv8 +
theme(legend.position="none")

ATP_turnover_box_plotv9

#Axis Options

ATP_turnover_box_plotv10 <- ATP_turnover_box_plotv9 +
theme(axis.text.x = element_text(size = 18, colour = "black"),
axis.text.y = element_text(size = 18, colour = "black"))

ATP_turnover_box_plotv10

ATP_turnover_box_plotv11 <- ATP_turnover_box_plotv10 +
theme(panel.background = element_rect(fill="white", colour="black"))
+ theme(panel.grid.major=element_line(colour="grey", linetype =
"dashed")) + theme(legend.title=element_blank())

ATP_turnover_box_plotv11

ATP_turnover_box_plotv12 <- ATP_turnover_box_plotv11 +
annotate("text", x = 0.6, y = 1200, label = "C", size=12, fontface=1)

tiff("Fig5C.tiff", width = 9, height = 5, units = 'in', res = 300)
plot(ATP_turnover_box_plotv12) # Make plot
dev.off()


#Fig. 5D

xorder <- c("Low", "Medium", "High")

ATP_Uptake_box_plot <-ggplot(ATP_Uptake, aes(Category, Value))

ATP_Uptake_box_plot


ATP_Uptake_box_plotv2 <- ATP_Uptake_box_plot + geom_boxplot(aes(fill
= Legend), outlier.colour = NA, notch=FALSE)

ATP_Uptake_box_plotv2


ATP_Uptake_box_plotv3 <- ATP_Uptake_box_plotv2 +
scale_x_discrete(limits=xorder)

ATP_Uptake_box_plotv3

ATP_Uptake_box_plotv4 <- ATP_Uptake_box_plotv3 +
scale_fill_manual(values=c("orangered", "lightgreen", "tan1" ))
```

```
ATP_Uptake_box_plotv4

ATP_Uptake_box_plotv5 <- ATP_Uptake_box_plotv4 + theme(panel.border =
element_rect(colour = "black", fill=NA, size=1))

ATP_Uptake_box_plotv5


ATP_Uptake_box_plotv6 <- ATP_Uptake_box_plotv5 +
theme(legend.title=element_blank(), axis.title.x =
element_text(size=18),axis.title.y=element_text(size=18))

ATP_Uptake_box_plotv6

ATP_Uptake_box_plotv7 <- ATP_Uptake_box_plotv6 +
labs(y=expression(ATP~uptake~~(nmol~ATP~L^{-1}~h^{-1})))

ATP_Uptake_box_plotv7

ATP_Uptake_box_plotv8 <- ATP_Uptake_box_plotv7 + xlab("Phosphate
Limitation")

ATP_Uptake_box_plotv8


ATP_Uptake_box_plotv9 <- ATP_Uptake_box_plotv8 +
theme(legend.position="none")

ATP_Uptake_box_plotv9

#Axis Options

ATP_Uptake_box_plotv10 <- ATP_Uptake_box_plotv9 + theme(axis.text.x =
element_text(size = 18, colour = "black"), axis.text.y =
element_text(size = 18, colour = "black"))

ATP_Uptake_box_plotv10

ATP_Uptake_box_plotv11 <- ATP_Uptake_box_plotv10 +
theme(panel.background = element_rect(fill="white", colour="black"))
+ theme(panel.grid.major=element_line(colour="grey", linetype =
"dashed")) + theme(legend.title=element_blank())

ATP_Uptake_box_plotv11

ATP_Uptake_box_plotv12 <- ATP_Uptake_box_plotv11 + annotate("text", x
= 0.6, y = 1.625, label = "D", size=12, fontface=1)

tiff("Fig5D.tiff", width = 9, height = 5, units = 'in', res = 300)
plot(ATP_Uptake_box_plotv12) # Make plot
dev.off()
```

**#Fig. 5E**

```
xorder <- c("Low", "Medium", "High")

DNA_turnover_box_plot <-ggplot(DNA_Turnover, aes(Category, Value))

DNA_turnover_box_plot
```

```
DNA_turnover_box_plotv2 <- DNA_turnover_box_plot +
geom_boxplot(aes(fill = Legend), outlier.colour = NA, notch=FALSE)

DNA_turnover_box_plotv2


DNA_turnover_box_plotv3 <- DNA_turnover_box_plotv2 +
scale_x_discrete(limits=xorder)

DNA_turnover_box_plotv3

DNA_turnover_box_plotv4 <- DNA_turnover_box_plotv3 +
scale_fill_manual(values=c("orangered", "lightgreen", "tan1" ))

DNA_turnover_box_plotv4

DNA_turnover_box_plotv5 <- DNA_turnover_box_plotv4 +
theme(panel.border = element_rect(colour = "black", fill=NA, size=1))

DNA_turnover_box_plotv5


DNA_turnover_box_plotv6 <- DNA_turnover_box_plotv5 +
theme(legend.title=element_blank(), axis.title.x =
element_text(size=18),axis.title.y=element_text(size=18))

DNA_turnover_box_plotv6

DNA_turnover_box_plotv7 <- DNA_turnover_box_plotv6 + ylab("DNA
turnover (h)")

DNA_turnover_box_plotv7

DNA_turnover_box_plotv8 <- DNA_turnover_box_plotv7 + xlab("Phosphate
Limitation")

DNA_turnover_box_plotv8


DNA_turnover_box_plotv9 <- DNA_turnover_box_plotv8 +
theme(legend.position="none")

DNA_turnover_box_plotv9

#Axis Options

DNA_turnover_box_plotv10 <- DNA_turnover_box_plotv9 +
theme(axis.text.x = element_text(size = 18, colour = "black"),
axis.text.y = element_text(size = 18, colour = "black"))

DNA_turnover_box_plotv10

DNA_turnover_box_plotv11 <- DNA_turnover_box_plotv10 +
theme(panel.background = element_rect(fill="white", colour="black"))
+ theme(panel.grid.major=element_line(colour="grey", linetype =
"dashed")) + theme(legend.title=element_blank())

DNA_turnover_box_plotv11
```

```
DNA_turnover_box_plotv12 <- DNA_turnover_box_plotv11 +
annotate("text", x = 0.6, y = 750, label = "E", size=12, fontface=1)

tiff("Fig5E.tiff", width = 9, height = 5, units = 'in', res = 300)
plot(DNA_turnover_box_plotv12) # Make plot
dev.off()
```

**#Fig. 5F**

```
xorder <- c("Low", "Medium", "High")

DNA_Uptake_box_plot <-ggplot(DNA_Uptake, aes(Category, Value))

DNA_Uptake_box_plot


DNA_Uptake_box_plotv2 <- DNA_Uptake_box_plot + geom_boxplot(aes(fill
= Legend), outlier.colour = NA, notch=FALSE)

DNA_Uptake_box_plotv2


DNA_Uptake_box_plotv3 <- DNA_Uptake_box_plotv2 +
scale_x_discrete(limits=xorder)

DNA_Uptake_box_plotv3

DNA_Uptake_box_plotv4 <- DNA_Uptake_box_plotv3 +
scale_fill_manual(values=c("orangered", "lightgreen", "tan1" ))

DNA_Uptake_box_plotv4

DNA_Uptake_box_plotv5 <- DNA_Uptake_box_plotv4 + theme(panel.border =
element_rect(colour = "black", fill=NA, size=1))

DNA_Uptake_box_plotv5


DNA_Uptake_box_plotv6 <- DNA_Uptake_box_plotv5 +
theme(legend.title=element_blank(), axis.title.x =
element_text(size=18),axis.title.y=element_text(size=18))

DNA_Uptake_box_plotv6

DNA_Uptake_box_plotv7 <- DNA_Uptake_box_plotv6 +
labs(y=expression(DNA~uptake~~(~mu~g~DNA~L^{-1}~h^{-1})))

DNA_Uptake_box_plotv7

DNA_Uptake_box_plotv8 <- DNA_Uptake_box_plotv7 + xlab("Phosphate
Limitation")

DNA_Uptake_box_plotv8


DNA_Uptake_box_plotv9 <- DNA_Uptake_box_plotv8 +
theme(legend.position="none")

DNA_Uptake_box_plotv9
```

```
#Axis Options

DNA_Uptake_box_plotv10 <- DNA_Uptake_box_plotv9 + theme(axis.text.x =
element_text(size = 18, colour = "black"), axis.text.y =
element_text(size = 18, colour = "black"))

DNA_Uptake_box_plotv10

DNA_Uptake_box_plotv11 <- DNA_Uptake_box_plotv10 +
theme(panel.background = element_rect(fill="white", colour="black"))
+ theme(panel.grid.major=element_line(colour="grey", linetype =
"dashed")) + theme(legend.title=element_blank())

DNA_Uptake_box_plotv11

DNA_Uptake_box_plotv12 <- DNA_Uptake_box_plotv11 + ylim(y=c(0,1))


DNA_Uptake_box_plotv13 <- DNA_Uptake_box_plotv12 + annotate("text", x
= 0.6, y = 0.85, label = "F", size=12, fontface=1)

tiff("Fig5F.tiff", width = 9, height = 5, units = 'in', res = 300)
plot(DNA_Uptake_box_plotv12) # Make plot
dev.off()
```
